# Fuzzy APPSS: A novel method for quantifying COVID-19 impact in India under triangular spherical fuzzy environment

**DOI:** 10.1038/s41598-024-82046-x

**Published:** 2024-12-28

**Authors:** Aicevarya Devi Sakthivel, Felix Augustin

**Affiliations:** Department of Mathematics, School of Advanced Sciences, Vellore Institute of Technology, 600127 Chennai, India

**Keywords:** Fuzzy APPSS method, Triangular spherical fuzzy number, Scoring function, Preference and performance, Diseases, Mathematics and computing

## Abstract

In the current scenario, decision-making models are essential for analyzing real-world problems. To address the dynamic nature of these problems, fuzzy decision-making models have been proposed by various researchers. However, an advanced technique is needed to assess uncertainty in real-time complex situations. Therefore, an association between preference and performance with satisfactory score (APPSS) method is introduced as a fuzzy decision-making method that incorporates two components: preference and performance. This method focuses on demonstrating a connection between preference and performance with a satisfactory measure. Preference analysis evaluates the significance of criteria, while performance analysis assesses the effectiveness of each alternative based on these criteria. Additionally, the satisfactory measure ensures the reliability of the outcomes. The applicability of the proposed method is demonstrated by analyzing the impact of COVID-19 on different age groups in India across various categories. The proposed method employs triangular spherical fuzzy numbers (TSFN), which is a mathematical model that extends beyond conventional fuzzy numbers by incorporating both triangular and spherical characteristics. Furthermore, a new scoring function for TSFN is developed using the graded mean integration method. The analysis reveals that the age group between 60-69 is highly vulnerable to COVID-19. The robustness of these outcomes is verified through sensitivity and comparative analyses. The findings also assist policymakers in more effectively assessing potential future health complications.

## Introduction

Decision-making involves selecting the optimal alternative from various possibilities, which may be based on a single criterion or multiple conflicting criteria. While single-criterion decisions are rare and straightforward, decisions involving multiple criteria are inherently complex. To manage this complexity, the multi-criteria decision-making (MCDM) approach is employed. This approach begins with a clear definition of the decision problem and objectives, resulting in a list of feasible alternatives and relevant criteria. A decision matrix is then constructed, with rows representing alternatives and columns representing criteria. Each cell in the matrix indicates how well an alternative performs relative to a criterion, using data gathered through expert judgment, experiments, surveys or historical records. The criteria are categorized into beneficiary and non-beneficiary to highlight positive contributions and negative impacts. The relative importance of the criteria in decision-making is determined through methods such as equal weighting (assigning equal importance to all criteria), direct rating (subjective weighting by experts) and pairwise comparison (comparing criteria against each other). When information is collected from multiple experts, an aggregation process is necessary. The aggregated outcomes are then used to rank the alternatives, facilitating the selection of the most optimal one.

In traditional decision-making models, the relationship between alternatives and criteria are treated as precise and deterministic. However, real-world scenarios often involve imprecise or uncertain information. To address this uncertainty, fuzzy logic was introduced by Zadeh^1^. It has been integrated into decision-making processes to develop the fuzzy MCDM models. The global impact of the COVID-19 pandemic has led to widespread health emergencies, creating disruptions in economies worldwide. These challenges have been analyzed by various researchers using fuzzy MCDM methods are provided below under various fuzzy environments like intuitionistic^2^, Pythagorean^3^, Fermatean^4^, picture^5^, spherical^6^, T-spherical^7^, etc.

In investigating the characteristics of COVID transmission, Ghorui et al.^8^ analyzed the influencing factors associated with COVID using analytic hierarchy process (AHP) and technique for order of preference by similarity to ideal solution (TOPSIS) within a hesitant fuzzy framework. The factors gathered from medical opinions, surveys and media sources include verbal spread, extended contact duration, lack of personal hygiene and social distancing, inadequate use of quality masks and transmission within hospitals and clinics are analyzed. TOPSIS is one of the most promising fuzzy MCDM methods was introduced by Hwang and Yoon^9^ in 1981, which scrutinize the minimum and maximum distances from the ideal and anti-ideal point. AHP was introduced by Satty^10^ and it is utilized for pairwise comparisons using a ratio scale to reveal the relationships between the compared elements. It scales exposes the connection in the comparisons and also incorporates a consistency check to detect and address any inconsistencies present in the judgments. The extended duration of contact emerged as a significant risk factor in the spread of COVID. Wan et al.^11^investigated the selection of hospitals for COVID patients using AHP and VIekriterijumsko KOmpromisno Rangiranje (VIKOR) methodologies under a trapezoidal interval type-2 fuzzy environment. VIKOR method was introduced by Opricovic^12^ highlights compromise solutions by integrating outranking and compromise-based decision-making approaches, considering both maximum group utility and minimum individual regret. The admission of a larger number of patients to hospitals results in insufficient medical equipment for effective treatment. So, Ozkan et al.^13^addressed the prioritization of high-risk COVID patients for admission to an intensive care unit using a combination of AHP and the multi-objective optimization method by ratio analysis (MOORA). MOORA was proposed by Brauers^14^ utilized a ratio analysis approach, where the evaluation of each alternative’s performance involved comparing its distance to both positive and negative ideal solutions. This methodology enabled a thorough assessment by considering both maximization and minimization criteria. COVID-19 situation posed numerous stressors for doctors as they continuously attended to patients. In a study by Fatima et al.^15^, the stress factors experienced by both private and government doctors were analyzed. And various alternatives were examined concerning stress factors including physical, ecological, mental, societal, traditional and emotional aspects, using the TOPSIS method. These factors were weighted through AHP. The findings revealed that doctors encountered challenges in balancing both personal and professional aspects of their lives during the pandemic. In an effort to alleviate the stress on doctors, Kang et al.^16^ examined the selection of medication service robots during the pandemic using the multi-attribute utility theory (MAUT) method. The pertinent criteria were identified through the Delphi method, and the selected criteria were assigned weights using the simplified best-worst method (SBWM).

In response to these factors, the lockdown was eased after three months with specific protocols. Devi et al.^17^ identified the significant protocol for controlling the spread of the coronavirus in India using the decision-making trial and evaluation laboratory (DEMATEL) approach under an intuitionistic fuzzy environment. The findings suggested that monitoring public well-being and controlling public movements were given a higher priority to control COVID. DEMATEL^18^ focused on the analysis of causal relationships among factors, providing a structured visual representation through a directed graph while addressing the interdependencies between these factors. Due to the prolonged lockdown, individuals faced numerous challenges. Stephen^19^ employed DEMATEL within a fuzzy inference system to identify the most significant criteria needed for diagnosing cardiovascular disease. Ahmad et al.^20^ conducted an analysis on various psychological factors that affected individuals during the pandemic using the TOPSIS method. The criteria such as severity, occurrence and detection methods were considered and weighted through the BWM. The anxiety and stress were identified as the most significant psychological elements that impacted a majority of individuals during the COVID pandemic. BWM^21^ helped in identifying the most important (best) and the least important (worst) criteria from the set of criteria. By capturing both extremes, it provided a comprehensive evaluation of the relative importance of all the criteria from best to worst. It also included a consistency check to ensure the reliability of the comparisons. The lockdown not only affected people’s lifestyles but also impacted the economies of national and international governments due to logistical interruptions. Ren et al.^22^ introduced a fuzzy MCDM approach based on regret theory using spherical fuzzy numbers (SFN). This theory helps to calculate the regret and rejoice values in performance of alternatives and criteria. Moreover, a distance measure called “Hellinger distance” was introduced for spherical fuzzy sets (SFS). All these concepts were applied to identify the best logistic providers. An analysis of the challenges faced by the supply chain during the pandemic was conducted by Magableh et al.^23^ using the TOPSIS method, and criteria being weighted through the analytic network process (ANP). It provided networked relationships among criteria and alternatives. Ali et al.^24^ analyzed the vaccine supply chain management using the TOPSIS approach and the robustness of the results was confirmed through the simple additive weighting (SAW) approach. Selerio et al.^25^ explored various emergency tasks of social media during disasters such as the COVID pandemic using DEMATEL and criteria are weighted using ANP. As the result, public health policies are prioritized, highlighting the measures that protect and improve the health and well-being of the public.

During the COVID pandemic, Ayyildiz et al.^26^ conducted an assessment to determine the optimal petrol station by considering factors such as accessibility, facility size, product range, safety measures and local population density. These factors were weighted using the AHP and the preferred petrol stations were scrutinized using the VIKOR method within a spherical fuzzy environment. Sharma et al.^27^ utilized the stepwise weight assessment ratio analysis (SWARA)^28^ method, an iterative process for adjusting weights, to analyze survival factors affecting supply chain management. SWARA method can revise the weights based on the ratios obtained during pairwise comparisons, allowing for a more flexible and fine-tuned evaluation of criteria. The tourism sector suffered major setbacks during the pandemic. In response, Hosseini et al.^29^ conducted an in-depth analysis of the factors influencing tourism recovery through the DEMATEL and VIKOR methods. Their research aimed to explore and evaluate the elements contributing to the revitalization of the tourism industry amid challenging circumstances. Besides tourism, the education sector also experienced significant disruptions during the pandemic. To enhance student learning, the conventional education system transitioned to online platforms. Aria et al.^30^ investigated the difficulties encountered by students in online education using the grey Delphi and DEMATEL methods. Data were collected from 86 students subjected to a questionnaire and 37 selected responses were further analyzed using the grey Delphi method to extract and identify significant challenging factors. Subsequently, these factors were analyzed using the grey DEMATEL method.

To eradicate the virus, countries collectively engaged in the pursuit of a vaccine. Several vaccines were emerged to combat the coronavirus. Meniz et al.^31^ conducted an analysis using AHP and VIKOR methods under interval type-2 fuzzy sets to determine the best vaccine among AstraZeneca, Biontech, Janssen, Moderna, Sinovac and Sputnik V. The findings revealed that the Sputnik V vaccine outperformed the other considered vaccines. Devi and Felix^32^ assessed ten COVID vaccines manufactured by various countries against COVID variants using the superiority and inferiority ranking (SIR) technique within the context of intuitionistic fuzzy-double parameters. The outcome indicated that the Moderna vaccine was the highest priority in combating the coronavirus. Deva et al.^33^ analyzed the various COVID vaccines using additive ratio assessment (ARAS) method under bipolar fuzzy graph, where the low, high, semi-perfect and perfect notations of bipolar fuzzy *p* competition graph were also introduced. After identifying the vaccines, Almulhim et al.^34^ prioritized the allocation of vaccines among specific groups, considering age, occupation, gender and existing health conditions. These alternatives were evaluated against various criteria using the complex proportional assessment (COPRAS) method under picture fuzzy context. COPRAS evaluates the relative importance of one criterion in relation to others, considering their proportional significance. The criteria were assigned weights using three different techniques: subjective, objective and integrated. The findings suggest that individuals with pre-existing health conditions are prioritized for vaccine allocation. Alsalema et al.^35^ scrutinized the recipients of the vaccine doses, employing the fuzzy-weighted zero-inconsistency (FWZIC) method to ascertain the importance of criteria. Additionally, utilized the fuzzy decision by opinion score method (FDOSM) to identify the most significant recipients within the context of T-spherical fuzzy. Ezhilarasan et al.^36^ introduced several defuzzification and ranking methods for bipolar heptagonal fuzzy numbers. The suggested methods was scrutinized for assessing vulnerability to stroke disease in different countries using the AHP and WASPAS methods. Khan et al.^37^ introduced the modified Hamming and Euclidean distances for the *q*-rung orthopair fuzzy context. Its suitability was validated in mass vaccination campaigns through the utilization of the VIKOR method. Swethaa et al.^38^ developed a ranking method for trapezoidal intuitionistic dense fuzzy numbers and applied for robot selection. Garai et al.^39^ scrutinized the finest COVID treatments for controlling the virus using the ranking interpreter technique under bipolar fuzzy analysis. Isolation emerged as the most effective treatment for controlling COVID. Apart from COVID-related research, various researchers have recently developed aggregation operators for different types of fuzzy numbers and verified their applicability in various decision-making techniques. Khan et al.^40^ introduced a novel approach utilizing *q*-rung orthopair fuzzy rough Dombi aggregation operators, incorporating Dombi norms and Dombi operational laws. These aggregation operators aided in integrating multiple expert judgements through methods such as extended TOPSIS, VIKOR and extended GRA. The effectiveness of these operators was validated through a construction company selection problem. Naeem et al.^41^ focused on developing various bipolar complex fuzzy Frank aggregation operators like power averaging, weighted averaging, ordered weighted averaging and geometric operators. The study also scrutinized combinations of these operators to effectively handle decision-making in two-dimensional (positive and negative) scenarios and was used to identify the optimal renewable energy sources among biomass, solar, wind and hydro. Garg et al.^42^ presented Aczel-Alsina power aggregation operators and applied to quantum computing within the framework of MCDM under a bipolar fuzzy environment. Senapati et al.^43^ proposed operators such as Dombi and Archimedean for *q*-rung orthopair fuzzy numbers, and their methodology was scrutinized using the candidate selection problem. Thilagavathy et al.^44 ^introduced aggregation operators such as Hamacher Heronian mean geometric, weighted geometric, ordered weighted geometric and hybrid geometric for T-spherical fuzzy numbers. These operators were validated using the lecturer selection problem through the simple multi-attribute rating technique (SMART) and TODIM methods to weight the criteria and rank the alternatives, respectively. Irvanizam and Zahara^45^ applied the single-valued trapezoidal neutrosophic number (SVTrNN) in the evaluation based on distance from average solution (EDAS) method to evaluate the quality of teachers. The EDAS method ranks alternatives based on their distance from an average solution. The positive and negative distance represents the best and worst possible alternatives, respectively. Zheng et al.^46^ proposed a MCDM approach based on the combined compromise solution (CoCoSo) method to diagnose Sepsis using interval-valued *q*-rung orthopair fuzzy sets (*q*-ROPFS). The evaluation is based on the aggregation operator and fuzzy entropy parameters of *q*-ROPFS. The effectiveness of the method was validated using numerical examples. Ranjbar et al.^47 ^utilized the AHP under a hesitant fuzzy environment to address the candidate selection problem. Irvanizam and Zahara^48^ enhanced the ranking of alternatives using the functional mapping of criterion sub-intervals into a single interval (RAFSI) method by integrating the SVTrNN to assess healthcare service quality. Also, the authors proposed the harmonic and arithmetic means for the SVTrNN. The harmonic mean is more influenced by smaller values because it emphasizes the reciprocals, while the arithmetic mean is more sensitive to larger values. In the study, various polyclinics were considered as alternatives which are evaluated based on criteria such as patient satisfaction level, quality of medical equipment, patient mortality rate, patient safety rate and level of interaction, with weights assigned using the ordinal priority approach. As the result, surgical oncology and otorhinolaryngology were given the highest priority. Irvanizam et al.^49^ developed the EDAS method using bipolar neutrosophic numbers to select small business groups. The bipolar neutrosophic set *N* in *A* consist of an element with its positive membership degrees $\bigl ($truth $\varrho _N^+ (a):A \rightarrow [0,1]$, indeterminacy $\varsigma _N^+ (a):A \rightarrow [0,1]$, falsity $\xi _N^+ (a):A \rightarrow [0,1]\bigl )$ and negative membership degrees $\bigl ($truth $\varrho _N^- (a):A \rightarrow [-1,0]$, indeterminacy $\varsigma _N^- (a):A \rightarrow [-1,0]$, falsity $\xi _N^- (a):A \rightarrow [-1,0]\bigl )$, where $\varrho _N^+,\varsigma _N^+ (a),\xi _N^+ (a) \in [0,1]$ and $\varrho _N^-,\varsigma _N^- (a),\xi _N^- (a) \in [-1,0]$. Thus, the bipolar neutrosophic numbers scrutinize the problems in both positive and negative aspects. Zia et al.^50^ proposed the concept of a complex linear Diophantine fuzzy set (CLDFS) to expand the solution space in MCDM by integrating reference parameters $\alpha$ and $\beta$. The study scrutinized two practical scenarios: bridge design selection and AI system choice to illustrate the practical impact and effectiveness of CLDFS in real-life applications. Lauron et al.^51^ analyzed African swine fever (ASF) using fuzzy AHP, VIKOR and DEMATEL methods. The fuzzy AHP assisted in obtaining information across various criteria. VIKOR was used to identify the most significant criteria, and DEMATEL determined the causal relationships among the identified criteria. This approach contributed to the development of effective ASF preventive measures. Demir et al.^52^ scrutinized various medical waste disposal methods using fuzzy compromise ranking of alternatives from distance to ideal solution (CRADIS) to rank the alternatives and fuzzy preference selection index (PSI) to weight the criteria. As the result, the autoclave emerged as the optimal disposal technique.

### Motivation and contributions

Through existing studies, it has been observed that fuzzy models and their integrated versions are commonly used to tackle COVID-related issues. Generally, fuzzy decision-making methods aim to rank various alternatives related to a given problem. However, the stability and consistency of these rankings have not been determined in existing methods. In these methods, alternatives are assessed using predefined criteria and individual approaches are employed to rank the alternatives and weight the criteria. The lack of stability and consistency in rankings and individual nature of ranking and weighting processes are the highlighted research gap. These constraints motivate the development of a novel fuzzy decision making model to effectively analyze the relationship between alternatives and criteria, ensuring a stable and consistent ranking process. Therefore, this study aims in designing a novel fuzzy model called association between preference and performance with satisfactory score (APPSS) using the Bart Kosko’s^53^ concept of bidirectional association memory (BAM) to evaluate relationship between the preference and performance with satisfactory conditions. The key novel contributions of the present study are detailed below.

- The association between preference and performance with satisfactory score (APPSS) method is designed to address the challenges in existing MCDM by incorporating fuzzy logic to handle preferences and performance evaluations. The model aims to: i) identify the weight of the criteria, ii) rank the alternatives and iii) assess the stability of the outcomes.
- Triangular spherical fuzzy numbers (TSFNs) address the uncertainty in assessing alternatives and criteria, effectively managing the aspects of belonging, non-belonging and neutrality degrees. By capturing these degrees, TSFNs achieve more informed and reliable decision-making.
- A scoring function is defined for TSFNs based on graded mean integration, which is utilized as a defuzzification technique in the proposed method.
- The BAM model is used to assess the relationship between alternatives and criteria by evaluating the performance of each alternative against the criteria based on the strength of their associations. Alternatives with stronger associations to positive criteria and weaker associations to negative criteria are considered to perform better, leading to more effective outcomes.
- Moreover, the model measures a satisfactory score to evaluate the stability of the outcomes. This score provides the consistency and reliability of the rankings under varying conditions.
- The applicability of the designed model is evaluated by scrutinizing the impact of COVID-19 on different age groups in India.
- The effectiveness of the proposed model is determined by comparing with other existing models and the sensitivity analysis is also performed by varying the input parameters to verify the robustness of the model.

The work is organized as follows: The “Preliminaries” section presents the basic concept of the proposed method. The “Proposed APPSS method” section introduces a novel fuzzy MCDM approach to evaluate preference and performance based on BAM with satisfactory scores. The “Illustration of the proposed method in finding the impact of COVID-19 in India” section demonstrates its applicability. The “Results and discussion” section discusses the obtained results, including comparative and sensitivity studies. Finally, the “Conclusion” section provides a summary of the manuscript.

## Preliminaries

### Definition 1

Let ${\tilde{Y}}^P$be the picture fuzzy set^5^ in *S* of the form ${\tilde{Y}}^P=\{(s,{\varrho }_{{\tilde{Y}}^P}(s),{\varsigma }_{{\tilde{Y}}^P}(s),{\xi }_{{\tilde{Y}}^P}(s))\ |\ s\in S\}$, where ${\varrho }_{{\tilde{Y}}^P}(s)$ is the belonging function defined by ${\varrho }_{{\tilde{Y}}^P}(s):S\rightarrow [0,1]$, ${\varsigma }_{{\tilde{Y}}^P}(s)$ is the neutral function defined by ${\varsigma }_{{\tilde{Y}}^P}(s):S\rightarrow [0,1]$ and ${\xi }_{{\tilde{Y}}^P}(s)$ is the non-belonging function defined by ${\xi }_{{\tilde{Y}}^P}(s):S\rightarrow [0,1]$ with the condition $0\le {\varrho }_{{\tilde{Y}}^P}(s)+{\varsigma }_{{\tilde{Y}}^P}(s)+{\xi }_{{\tilde{Y}}^P}(s)\le 1$ for every $s\in S$. Additionally, ${\flat }_{{\tilde{Y}}^P}(s)$ is the refusal function of *s* in ${{\tilde{Y}}^P}$ determined by ${\flat }_{{\tilde{Y}}^P}(s)=1-({\varrho }_{{\tilde{Y}}^P}(s)+{\varsigma }_{{\tilde{Y}}^P}(s)+{\xi }_{{\tilde{Y}}^P}(s))$.

### Definition 2

**Fig. 1:**
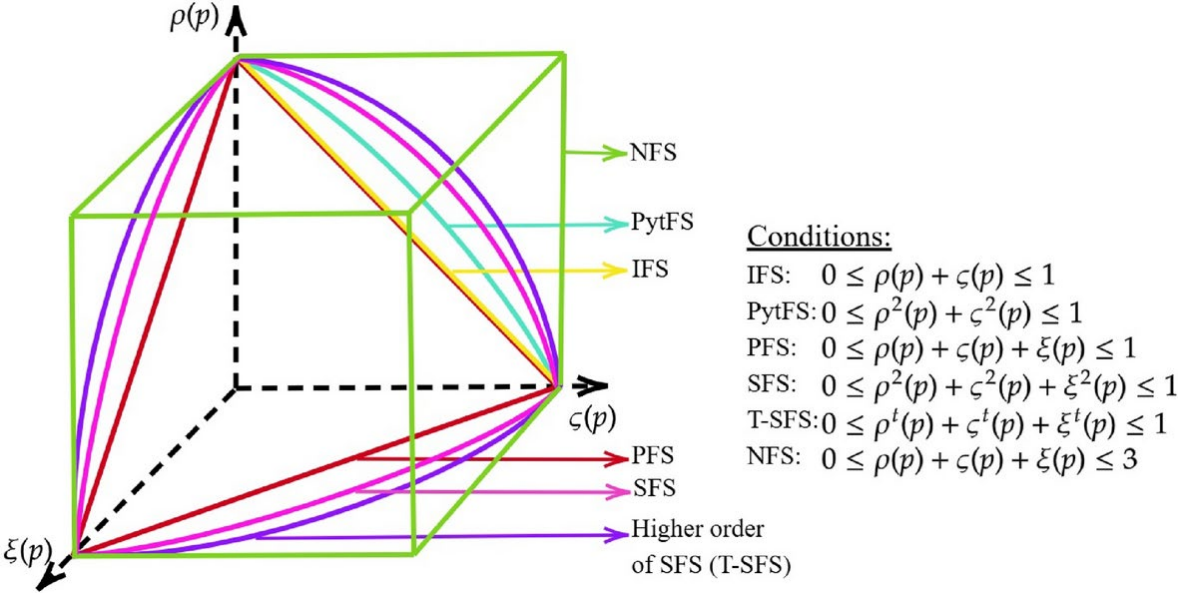
Representation of different types of extended fuzzy sets.

Let ${\tilde{Y}}^S$be the spherical fuzzy set^6^ in *P* of the form ${\tilde{Y}}^S=\{(p,{\varrho }_{{\tilde{Y}}^S}(p),{\varsigma }_{{\tilde{Y}}^S}(p),{\xi }_{{\tilde{Y}}^S}(p))\ |\ p\in P\}$, where ${\varrho }_{{\tilde{Y}}^S}(p)$ is the belonging function defined by ${\varrho }_{{\tilde{Y}}^S}(p):P\rightarrow [0,1]$, ${\varsigma }_{{\tilde{Y}}^S}(p)$ is the neutral function defined by ${\varsigma }_{{\tilde{Y}}^S}(p):P\rightarrow [0,1]$ and ${\xi }_{{\tilde{Y}}^S}(p)$ is the non-belonging function defined by ${\xi }_{{\tilde{Y}}^S}(p):P\rightarrow [0,1]$ with the condition $0\le {\big ({\varrho }_{{\tilde{Y}}^S}(p)\big )}^{2}+{{\big (\varsigma }_{{\tilde{Y}}^S}(p)\big )}^{2}+{{\big (\xi }_{{\tilde{Y}}^S}(p)\big )}^{2}\le 1$ for every $p\in P$. Additionally, ${\flat }_{{\tilde{Y}}^S}(p)$ is the refusal function of *p* in ${{\tilde{Y}}^S}$ determined by ${\flat }_{{\tilde{Y}}^S}(p)=\sqrt{1-\Big ({\big ({\varrho }_{{\tilde{Y}}^S}(p)\big )}^{2}+{\big ({\varsigma }_{{\tilde{Y}}^S}(p)\big )}^{2}+{\big ({\xi }_{{\tilde{Y}}^S}(p)\big )}^{2}\Big )}$. The diagrammatic representation of TSFS is presented in Figure 1.

**Proposed definitions**

By integrating the characteristics of triangular fuzzy number and spherical fuzzy number, a triangular spherical fuzzy number (TSFN) is designed.

### Definition 3

A TSFN on the real line $\mathbb {R}$ is represented as ${\tilde{Y}}^{TS}=\bigl \{\big ((a_1,b,c_1);\Im _1\big ),\big ((a_2,b,c_2);\Im _2\big ),\big ((a_3,b,c_3);\Im _3\big )\bigl \}$. Then, its belonging, neutral and non-belonging functions are defined as follows.

$$\begin{aligned} \varrho _{\widetilde{Y}^{TS}}(y)={\left\{ \begin{array}{ll} \frac{\Im _1(y-a_1)}{b-a_1}, & a_1 \le y< b \\ \Im _1, & y=b\\ \frac{1(c_1-y)}{c_1-b}, & b< y \le c_1\\ 0, & otherwise \end{array}\right. }\\ \varsigma _{\widetilde{Y}^{TS}}(y)={\left\{ \begin{array}{ll} \frac{(b-y)+\Im _2(y-a_2)}{b-a_2}, & a_2 \le y< b \\ \Im _2, & y=b\\ \frac{(y-b)+\Im _2(c_2-y)}{c_2-b}, & b< y \le c_2\\ 0, & otherwise \end{array}\right. }\\ \xi _{\widetilde{Y}^{TS}}(y)={\left\{ \begin{array}{ll} \frac{(b-y)+\Im _3(y-a_3)}{b-a_3}, & a_3 \le y< b \\ \Im _3, & y=b\\ \frac{(y-b)+\Im _3(c_3-y)}{c_3-b}, & b < y \le c_3\\ 0, & otherwise \end{array}\right. } \end{aligned}$$

where $$0 \le {\big (\varrho _{\widetilde{Y}^{TS}}(y)\big )}^{2} + {\big (\varsigma _{\widetilde{Y}^{TS}}(y)\big )}^{2} + {\big (\xi _{\widetilde{Y}^{TS}}(y)\big )}^{2} \le 1$$.

**Remark 3.1:** TSFN can be denoted as $\tilde{Y}^{TS}=\bigl \{(a,b,c);\varrho _{\tilde{Y}^{TS}}(y),\varsigma _{\tilde{Y}^{TS}}(y),\xi _{\tilde{Y}^{TS}}(y)\bigl \}$ when $(a_1,b,c_1)=(a_2,b,c_2)=(a_3,b,c_3)$.

Here, *b* indicates the mean value, $a=a_1=a_2=a_3$ and $c=c_1=c_2=c_3$ are the left and right spreads, respectively.

### Definition 4

Let $\tilde{Y}_{1}^{TS}=\Bigl \{(a_1,b_1,c_1);\varrho _{\tilde{Y}_{1}^{TS}},\varsigma _{\tilde{Y}_{1}^{TS}},\xi _{\tilde{Y}_{1}^{TS}}\Bigl \}$ and $\tilde{Y}_{2}^{TS}=\Bigl \{(a_2,b_2,c_2);\varrho _{\tilde{Y}_{2}^{TS}},\varsigma _{\tilde{Y}_{2}^{TS}},\xi _{\tilde{Y}_{2}^{TS}}\Bigl \}$ be two TSFNs, then its arithmetic operations are as follows.

- $$\tilde{Y}_{1}^{TS} + \tilde{Y}_{2}^{TS} = \Bigl (\bigl (a_1+a_2, \ b_1+b_2, \ c_1+c_2\bigl ); \ \varrho _{\tilde{Y}_{1}^{TS}}+\varrho _{\tilde{Y}_{2}^{TS}}-\varrho _{\tilde{Y}_{1}^{TS}}\varrho _{\tilde{Y}_{2}^{TS}}, \ \varsigma _{\tilde{Y}_{1}^{TS}}\varsigma _{\tilde{Y}_{2}^{TS}}, \ \xi _{\tilde{Y}_{1}^{TS}}\xi _{\tilde{Y}_{2}^{TS}}\Bigl )$$
- $$\tilde{Y}_{1}^{TS} \times \tilde{Y}_{2}^{TS} = \Bigl (\bigl (a_1a_2, \ b_1b_2, \ c_1c_2\bigl ); \varrho _{\tilde{Y}_{1}^{TS}}\varrho _{\tilde{Y}_{2}^{TS}}, \ \varsigma _{\tilde{Y}_{1}^{TS}} + \varsigma _{\tilde{Y}_{2}^{TS}} -\varsigma _{\tilde{Y}_{1}^{TS}}\varsigma _{\tilde{Y}_{2}^{TS}}, \ \xi _{\tilde{Y}_{1}^{TS}} + \xi _{\tilde{Y}_{2}^{TS}} -\xi _{\tilde{Y}_{1}^{TS}}\xi _{\tilde{Y}_{2}^{TS}}\Bigl )$$
- $$\lambda \tilde{Y}_{1}^{TS} = \Bigl ( \bigl (\lambda a_1, \ \lambda b_1, \ \lambda c_1 \bigl ); \ 1-{\bigl (1-\varrho _{\tilde{Y}_{1}^{TS}}\bigl )}^{\lambda }, \ {\bigl (\varsigma _{\tilde{Y}_{1}^{TS}}\bigl )}^{\lambda }, \ {\bigl (\xi _{\tilde{Y}_{1}^{TS}}\bigl )}^{\lambda } \Bigl ), \ \lambda \ge 0$$
- $$\begin{aligned}{{(\tilde{Y}_{1}^{TS})}}^{\lambda } = \Bigl ( \bigl ( {a_1}^{\lambda }, \ {b_1}^{\lambda }, \ {c_1}^{\lambda } \bigl ); {\bigl (\varrho _{\tilde{Y}_{1}^{TS}}\bigl )}^{\lambda }, \ {1-\bigl (1-\varsigma _{\tilde{Y}_{1}^{TS}}\bigl )}^{\lambda }, {1-\bigl (1-\xi _{\tilde{Y}_{1}^{TS}}\bigl )}^{\lambda } \Bigl ), \ \lambda \ge 0 \end{aligned}$$

The subtraction and division operators for TSFN are developed based on the article^54^.

### Definition 5

Subtraction and Division operations for TSFN

For the two TSFN $\tilde{Y}_{1}^{TS}=\Bigl \{(a_1,b_1,c_1);\varrho _{\tilde{Y}_{1}^{TS}},\varsigma _{\tilde{Y}_{1}^{TS}},\xi _{\tilde{Y}_{1}^{TS}}\Bigl \}$ and $\tilde{Y}_{2}^{TS}=\Bigl \{(a_2,b_2,c_2);\varrho _{\tilde{Y}_{2}^{TS}},\varsigma _{\tilde{Y}_{2}^{TS}},\xi _{\tilde{Y}_{2}^{TS}}\Bigl \}$, the subtraction operation is defined as

$$\begin{aligned} \tilde{Y}_{1}^{TS} - \tilde{Y}_{2}^{TS} = \Biggl \{\Big (a_1 - c_2, b_1 - b_2, c_1 - a_2\Big ); \frac{\varrho _{\tilde{Y}_{1}^{TS}} - \varrho _{\tilde{Y}_{2}^{TS}}}{1-\varrho _{\tilde{Y}_{2}^{TS}}}, \ \frac{\varsigma _{\tilde{Y}_{1}^{TS}}}{\varsigma _{\tilde{Y}_{2}^{TS}}}, \ \frac{\xi _{\tilde{Y}_{1}^{TS}}}{\xi _{\tilde{Y}_{2}^{TS}}} \Biggl \} \end{aligned}$$

which holds true under the specified conditions $\tilde{Y}_{1}^{TS}> \tilde{Y}_{2}^{TS}, \ \varrho _{\tilde{Y}_{2}^{TS}} \ne 1, \ \varsigma _{\tilde{Y}_{2}^{TS}} \ne 0, \ \xi _{\tilde{Y}_{2}^{TS}} \ne 0$.

The division operation is defined as

$$\begin{aligned} \tilde{Y}_{1}^{TS} / \tilde{Y}_{2}^{TS} = \Biggl \{\Big (a_1 - c_2, b_1 - b_2, c_1 - a_2\Big ); \frac{\varrho _{\tilde{Y}_{1}^{TS}}}{\varrho _{\tilde{Y}_{2}^{TS}}}, \ \frac{\varsigma _{\tilde{Y}_{1}^{TS}} - \varsigma _{\tilde{Y}_{2}^{TS}}}{1 - \varsigma _{\tilde{Y}_{2}^{TS}}}, \ \frac{\xi _{\tilde{Y}_{1}^{TS}} - \xi _{\tilde{Y}_{2}^{TS}}}{1 - \xi _{\tilde{Y}_{2}^{TS}}} \Biggl \} \end{aligned}$$

which holds true under the specified conditions $\tilde{Y}_{2}^{TS}> \tilde{Y}_{1}^{TS}, \ \varrho _{\tilde{Y}_{2}^{TS}} \ne 0, \ \varsigma _{\tilde{Y}_{2}^{TS}} \ne 1, \ \xi _{\tilde{Y}_{2}^{TS}} \ne 1$.

### Definition 6

Let $\widetilde{U}=\big ((a,b,c);\varrho (u),\varsigma (u),\xi (u) \big )$ be the TSFN in *P*. Using the graded mean integration, the scoring function $Sco(\widetilde{U}):SFS(P) \rightarrow [-1,1]$ is defined below.

1 $$\begin{aligned} {Sco^{TS}}({\widetilde{U}})=2 \Big (\frac{a}{12}+\frac{b}{3}+\frac{c}{12}\Big ) \times \Big ( \frac{\varrho (u) + \varsigma (u) + \xi (u)}{3}\Big ) \ \forall \ Sco^{TS}({\widetilde{U}}) \in SFS(P) \end{aligned}$$

### Definition 7

Let $\widetilde{U}_{1} = \big ((a_{1},b_{1},c_{1});\varrho _{1}(u),\varsigma _{1}(u),\xi _{1}(u)\big )$ and $\widetilde{U}_{2} = \big ((a_{2},b_{2},c_{2});\varrho _{2}(u),\varsigma _{2}(u),\xi _{2}(u)\big )$ be the two TSFN, then the score of $\widetilde{U}_1$ and $\widetilde{U}_2$ are determined through the score function in Equation (1). The possible score conditions are given below.

- $$Sco(\widetilde{U}_1)> Sco(\widetilde{U}_2)$$, if $$\varrho _{\widetilde{U}_1}(u)> \varrho _{\widetilde{U}_2}(u), \varsigma _{\widetilde{U}_1}(u)> \varsigma _{\widetilde{U}_2}(u), \xi _{\widetilde{U}_1}(u) < \xi _{\widetilde{U}_2}(u)$$
- $$Sco(\widetilde{U}_2)> Sco(\widetilde{U}_1)$$, if $$\varrho _{\widetilde{U}_1}(u)< \varrho _{\widetilde{U}_2}(u), \varsigma _{\widetilde{U}_1}(u) < \varsigma _{\widetilde{U}_2}(u), \xi _{\widetilde{U}_1}(u)> \xi _{\widetilde{U}_2}(u)$$
- $$Sco(\widetilde{U}_1) = Sco(\widetilde{U}_2)$$, if $$\varrho _{\widetilde{U}_1}(u) = \varrho _{\widetilde{U}_2}(u), \varsigma _{\widetilde{U}_1}(u) = \varsigma _{\widetilde{U}_2}(u), \xi _{\widetilde{U}_1}(u) = \xi _{\widetilde{U}_2}(u)$$

### Definition 8

Weighted aggregation operator for TSFN: Let $\widetilde{U}_i = \{(a_i,b_i,c_i); \varrho _{i}, \varsigma _{i}, \xi _{i}\}, \ i=1,2,\ldots ,m$ be the TSFNs and corresponding weights are $W = [w_1 \ w_2 \ \cdots \ w_m]$ and $\sum \limits _{i=1}^{m} w_{i} =1$ then

$$\begin{aligned} Agg \bigl (\widetilde{U}_1, \widetilde{U}_2,\cdots ,\widetilde{U}_m \bigl ) = \Bigl ( \bigl ( \sum \limits _{i=1}^{m} w_ia_i, \sum \limits _{i=1}^{m} w_ib_i, \sum \limits _{i=1}^{m} w_ic_i \big ); 1 - \prod \limits _{i=1}^{m} (1- \varrho _{i}^{w_i}), \prod \limits _{i=1}^{m} \varsigma _{i}^{w_i}, \prod \limits _{i=1}^{m} \xi _{i}^{w_i} \Bigl ) \end{aligned}$$

Let $$m=2$$, then

$$\begin{aligned} & Agg \bigl (\widetilde{U}_1, \widetilde{U}_2 \bigl ) = \Bigl ( \bigl ( (a_1,b_1,c_1); \varrho _1, \varsigma _1, \xi _1 \bigl ) + \bigl ( (a_2,b_2,c_2); \varrho _2, \varsigma _2, \xi _2 \bigl ) \Bigl )\\ & \quad =\Bigl ( \bigl (w_1a_1 + w_2a_2, w_1b_1 + w_2b_2, w_1c_1 + w_2c_2\bigl ); 1- {(1-\varrho _1)}^{w_1} + 1- {(1-\varrho _2)}^{w_2} - \bigl ( {(1-\varrho _1)}^{w_1} {(1-\varrho _2)}^{w_2} \bigl ),\\ & \quad \bigl (\varsigma _{1}^{w_1}\varsigma _{2}^{w_2}\bigl ) \bigl (\xi _{1}^{w_1}\xi _{2}^{w_2}\bigl ) \Bigl ) \end{aligned}$$

Now, $$m=n$$

$$Agg \bigl (\widetilde{U}_1, \widetilde{U}_2,\cdots ,\widetilde{U}_n \bigl ) = \Bigl ( \bigl ( \sum \limits _{i=1}^{n} w_ia_i, \sum \limits _{i=1}^{n} w_ib_i, \sum \limits _{i=1}^{n} w_ic_i \big ); 1 - \prod \limits _{i=1}^{n} (1- \varrho _{i}^{w_i}), \prod \limits _{i=1}^{n} \varsigma _{i}^{w_i}, \prod \limits _{i=1}^{n} \xi _{i}^{w_i} \Bigl )$$.

### Definition 9

Mean and standard deviation of TSFN

The mean is the arithmetic average of a set of values. It is calculated by summing all the values in the dataset and dividing by the total number of values. The mean provides a measure of central tendency, which provides the “average” value in a dataset.

$$\begin{aligned} \text {Mean} \ (\bar{x}) = \frac{\sum \limits _{i=1}^{n} x_i}{n} \end{aligned}$$

where $x_i=\big ((a_i,b_i,c_i); \ \varrho _i, \ \varsigma _i, \ \xi _i \big ), \ i=1,2,\ldots ,n,$ *n* is the total number of elements in the set.

The standard deviation measures the spread or dispersion of a dataset relative to its mean.

$$\begin{aligned} \text {Standard deviation} \ (\sigma ) = \sqrt{\frac{\sum \limits _{i=1}^{n} \big (x_i - \bar{x}\big )}{n-1}} \end{aligned}$$

where $x_i=\big ((a_i,b_i,c_i); \ \varrho _i, \ \varsigma _i, \ \xi _i \big ), \ i=1,2,\ldots ,n,$ *n* is the total number of elements in the set.

## Proposed APPSS method

A comprehensive and interactive decision-making approach, named the association between preference and performance with satisfactory score (APPSS) has been devised by utilizing fuzzy logic to proficiently rank alternatives. The process involves three distinct segments, incorporating various alternatives ($\mathfrak {d_a}$), where $\mathfrak {a}=1,2,\ldots ,f$ and related criteria ($\mathfrak {C_b}$), where $\mathfrak {b}=1,2,\ldots ,g$ are framed with the input of field experts ($\mathcal {E}_c$), where $c=1,2,\ldots ,e$.

**Segment I: Preference score**

**Step 1:** Collect the information of criteria.

The information about the local and global criteria are collected.

**Step 2:** Construct the pairwise linguistic preference matrix (PLPM).

For the considered local criteria $\mathfrak {C_b}$, the PLPM ${H}={[{h}_{\mathfrak {t}\mathfrak {b}}]}_{g \times g}, \ \text {where} \ \mathfrak {t},\mathfrak {b}=1,2,\ldots ,g \ \text {and} \ \mathfrak {t} \ne \mathfrak {b}$, is constructed using linguistic terms.

**Step 3:** Convert the PLPM into triangular spherical fuzzy matrix (TSFM).

The PLPM is transmuted into the TSFM using the linguistic terms.where $\tilde{h}_{\mathfrak {t}\mathfrak {b}}=\big ((x_{\mathfrak {t}\mathfrak {b}}, y_{\mathfrak {t}\mathfrak {b}}, z_{\mathfrak {t}\mathfrak {b}}); \ell _{\mathfrak {t}\mathfrak {b}}(h), \wp _{\mathfrak {t}\mathfrak {b}}(h), \partial _{\mathfrak {t}\mathfrak {b}}(h) \big ); \ \mathfrak {t},\mathfrak {b}=1,2,\ldots ,g \ \text {and} \ \mathfrak {t} \ne \mathfrak {b}$.

**Step 4:** Determine the reference point.

The mean value is calculated for TSFM to measure the central tendency.

2 $$\begin{aligned} M^{P}=\frac{\sum \limits _{\mathfrak {b}=1}^{g} \tilde{h}_{\mathfrak {t}\mathfrak {b}}}{g} \end{aligned}$$

where $$\tilde{h}_{\mathfrak {t}\mathfrak {b}}=\big ((x_{\mathfrak {t}\mathfrak {b}}, y_{\mathfrak {t}\mathfrak {b}}, z_{\mathfrak {t}\mathfrak {b}}); \ell _{\mathfrak {t}\mathfrak {b}}(h), \wp _{\mathfrak {t}\mathfrak {b}}(h), \partial _{\mathfrak {t}\mathfrak {b}}(h) \big ); \ \mathfrak {t},\mathfrak {b}=1,2,\ldots ,g \ \text {and} \ \mathfrak {t} \ne \mathfrak {b}$$.

$$\begin{aligned} \ \ \ \ \ \ \ \mathfrak {C}_1 \ \mathfrak {C}_2 \ \cdots \ \mathfrak {C}_g \\ M^{P}=[\tilde{m}_1 \ \tilde{m}_2 \ \cdots \ \tilde{m}_g] \end{aligned}$$

where $$\tilde{m}_b = \big ((x_{\mathfrak {b}}, y_{\mathfrak {b}}, z_{\mathfrak {b}}); \ell _{\mathfrak {b}}(h), \wp _{\mathfrak {b}}(h), \partial _{\mathfrak {b}}(h) \big ); \ \mathfrak {b}=1,2,\ldots ,g.$$

**Step 5:** Calculate the standard deviation.

The standard deviation is calculated to measure the dispersion of each criterion in TSFM.

3 $$\begin{aligned} SD^{P}=\sqrt{\frac{1}{g-1} \sum \limits _{\begin{array}{c} \mathfrak {t},\mathfrak {b}=1 \\ \mathfrak {t} \ne \mathfrak {b} \end{array}}^{g} {\big (\tilde{h}_{\mathfrak {t}\mathfrak {b}}-M^{P}\big )}^2} \end{aligned}$$

$$\begin{aligned} \ \ \ \ \ \ \ \ \ \ \ \mathfrak {C}_1 \ \mathfrak {C}_2 \ \cdots \ \mathfrak {C}_g \\ SD^{P}=[\tilde{s}_1 \ \tilde{s}_2 \ \cdots \ \tilde{s}_g] \end{aligned}$$

where $$\tilde{s}_b = \big ((x_{\mathfrak {b}}, y_{\mathfrak {b}}, z_{\mathfrak {b}}); \ell _{\mathfrak {b}}(h), \wp _{\mathfrak {b}}(h), \partial _{\mathfrak {b}}(h) \big ); \ \mathfrak {b}=1,2,\ldots ,g.$$

**Step 6:** Demonstrate the standard matrix.

The standard matrix (${\widetilde{SM}}^P$) is measured using Equation (4).

4 $$\begin{aligned} {\widetilde{SM}}^P=\frac{\tilde{h}_{\mathfrak {t}\mathfrak {b}}-M^{P}}{SD^{P}} \end{aligned}$$

where $${\widetilde{SM}}^P={[{\tilde{s}}_{\mathfrak {t}\mathfrak {b}}]}_{g \times g}; \ {\tilde{s}}_{\mathfrak {t}\mathfrak {b}}= \big ((x_{\mathfrak {t}\mathfrak {b}}, y_{\mathfrak {t}\mathfrak {b}}, z_{\mathfrak {t}\mathfrak {b}}); \ell _{\mathfrak {t}\mathfrak {b}}(s), \wp _{\mathfrak {t}\mathfrak {b}}(s), \partial _{\mathfrak {t}\mathfrak {b}}(s) \big ); \ \mathfrak {t},\mathfrak {b}=1,2,\ldots ,g \ \text {and} \ \mathfrak {t} \ne \mathfrak {b}$$.

**Step 7:** Determine the normalized matrix (${\widetilde{NM}}^P$).

The ${\widetilde{SM}}^P$ is normalized using Equation (5).

5 $$\begin{aligned} {\widetilde{NM}}^P=\frac{\tilde{s}_{\mathfrak {t}\mathfrak {b}}- \min (\tilde{s}_{\mathfrak {t}\mathfrak {b}})}{\max (\tilde{s}_{\mathfrak {t}\mathfrak {b}}) - \min (\tilde{s}_{\mathfrak {t}\mathfrak {b}})}, \ \text {where}\ \mathfrak {t},\mathfrak {b}=1,2,\ldots ,g \ \text {and} \ \mathfrak {t} \ne \mathfrak {b}. \end{aligned}$$

where $${\widetilde{NM}}^P={[{\tilde{n}}_{\mathfrak {t}\mathfrak {b}}]}_{g \times g}; \ {\tilde{n}}_{\mathfrak {t}\mathfrak {b}}= \big ((x_{\mathfrak {t}\mathfrak {b}}, y_{\mathfrak {t}\mathfrak {b}}, z_{\mathfrak {t}\mathfrak {b}}); \ell _{\mathfrak {t}\mathfrak {b}}(n), \wp _{\mathfrak {t}\mathfrak {b}}(n), \partial _{\mathfrak {t}\mathfrak {b}}(n) \big ); \ \mathfrak {t},\mathfrak {b}=1,2,\ldots ,g \ \text {and} \ \mathfrak {t} \ne \mathfrak {b}$$; $${\widetilde{NM}}^P \in [0,1]$$ 

**Step 8:** Determine the defuzzified matrix.

In the normalized matrix ($\widetilde{NM}^P$), all the entries are in the TSFN. So, the $\widetilde{NM}^P$ is defuzzified to obtain the crisp score using the proposed scoring function in Equation (6).

6 $$\begin{aligned} {O^{P}}=2 \Big (\frac{x_{\mathfrak {t}\mathfrak {b}}}{12}+\frac{y_{\mathfrak {t}\mathfrak {b}}}{3}+\frac{z_{\mathfrak {t}\mathfrak {b}}}{12}\Big ) \times \Big ( \frac{\ell _{\mathfrak {t}\mathfrak {b}} + \wp _{\mathfrak {t}\mathfrak {b}} + \partial _{\mathfrak {t}\mathfrak {b}}}{3}\Big ) \end{aligned}$$

where $${{O}^{P}}={[{{o}}_{\mathfrak {t}\mathfrak {b}}]}_{{g \times g}}=[{o}_{\mathfrak {t}\mathfrak {b}}$$]; $$\mathfrak {t},\mathfrak {b}=1,2,\ldots ,g \ \text {and} \ \mathfrak {t} \ne \mathfrak {b}$$.

When dealing with multiple decision matrices, the aggregation process is necessary. The defuzzified matrices are aggregated to convert into single preference matrix.

7 $$\begin{aligned} {Q}=\frac{\sum \limits _{c=1}^{e} {o}_{\mathfrak {t}\mathfrak {b}}^{c}}{e} \end{aligned}$$

where $${{Q}}={[{{q}}_{\mathfrak {t}\mathfrak {b}}]}_{g \times g}; \ [{q}_{\mathfrak {t}\mathfrak {b}}]$$; $$\mathfrak {t},\mathfrak {b}=1,2,\ldots ,g \ \text {and} \ \mathfrak {t} \ne \mathfrak {b}$$.

**Step 9:** Determine the weight of local criteria.

The summation value of each local criteria is determined using Equation (8).

8 $$\begin{aligned} LC(\mathfrak {C_b})=\sum \limits _{\begin{array}{c} \mathfrak {t},\mathfrak {b}=1 \\ \mathfrak {t} \ne \mathfrak {b} \end{array}}^{g} {o}_{\mathfrak {t}\mathfrak {b}} \end{aligned}$$

**Step 10:** Determine the weight of global criteria.

The global criteria weights are determined by aggregating their respective local criteria values.

9 $$\begin{aligned} GC(\mathfrak {C_b})=\frac{\sum \limits _{\mathfrak {b}=1}^{g} LC(\mathfrak {C_b})}{LC(\mathfrak {C_b})} \end{aligned}$$

**Step 11:** Determine the overall score.

The overall score of each local criteria is evaluated using weighted average in Equation (10).

10 $$\begin{aligned} \mathfrak {C_b}=\frac{\big (LC(\mathfrak {C_b})\big ) \alpha + \big (GC(\mathfrak {C_b})\big ) (1-\alpha )}{2}, \ \text {where} \ \alpha \in [0,1]. \end{aligned}$$

**Step 12:** Determine the preference vector.

The preference of each local criteria are determined using Equation (11).

11 $$\begin{aligned} \mathfrak {C_b}=\frac{\mathfrak {C_b}}{\sum \limits _{\mathfrak {b}=1}^{g} \mathfrak {C_b}} \end{aligned}$$

Therefore, the final preference vector is $W=[\mathfrak {C}_1 \ \mathfrak {C}_2 \ \cdots \ \mathfrak {C}_g]$.

**Segment II: Performance score**

**Step 1:** Collect the information. For the considered alternatives $\mathfrak {d_a}$ and criteria $\mathfrak {C_b}$, where $\mathfrak {a}=1,2,\ldots ,f; \ \mathfrak {b}=1,2,\ldots ,g$, the relations are considered from real data or from experts $\mathcal {E}_c$, where $c=1,2,\ldots ,e$.

**Step 2:** Construct the linguistic performance matrix (LPeM).

The LPeM ${L}={[{L}_{\mathfrak {a}\mathfrak {b}}]}_{f \times g}, \ \text {where} \ \mathfrak {a}=1,2,\ldots ,f;\ \mathfrak {b}=1,2,\ldots ,g$, is constructed with the help of experts.

**Step 3:** Convert the LPeM into TSFM. The LPeM is converted into TSFM using the linguistic scale.where $\tilde{l}_{\mathfrak {a}\mathfrak {b}}=\big ((x_{\mathfrak {a}\mathfrak {b}}, y_{\mathfrak {a}\mathfrak {b}}, z_{\mathfrak {a}\mathfrak {b}}); \ell _{\mathfrak {a}\mathfrak {b}}(l), \wp _{\mathfrak {a}\mathfrak {b}}(l), \partial _{\mathfrak {a}\mathfrak {b}}(l) \big ); \ a=1,2,\ldots ,f; \ b=1,2,\ldots ,g$.

**Step 4:** Determine the reference point. The mean value is calculated for TSFM to measure the central tendency.

12 $$\begin{aligned} M^{Pe}=\frac{\sum \limits _{\mathfrak {b}=1}^{g} \tilde{l}_{\mathfrak {a}\mathfrak {b}}}{f} \end{aligned}$$

where $$\tilde{l}_{\mathfrak {a}\mathfrak {b}}=\big ((x_{\mathfrak {a}\mathfrak {b}}, y_{\mathfrak {a}\mathfrak {b}}, z_{\mathfrak {a}\mathfrak {b}}); \ell _{\mathfrak {a}\mathfrak {b}}(l), \wp _{\mathfrak {a}\mathfrak {b}}(l), \partial _{\mathfrak {a}\mathfrak {b}}(l) \big ); \ \mathfrak {a}=1,2,\ldots ,f, \ \mathfrak {b}=1,2,\ldots ,g.$$

$$\begin{aligned} \ \ \ \ \ \ \ \mathfrak {C}_1 \ \mathfrak {C}_2 \ \cdots \ \mathfrak {C}_g \\ M^{Pe}=[\tilde{m}_1 \ \tilde{m}_2 \ \cdots \ \tilde{m}_g] \end{aligned}$$

where $$\tilde{m}_b = \big ((x_{\mathfrak {b}}, y_{\mathfrak {b}}, z_{\mathfrak {b}}); \ell _{\mathfrak {b}}(h), \wp _{\mathfrak {b}}(h), \partial _{\mathfrak {b}}(h) \big ); \ \mathfrak {b}=1,2,\ldots ,g.$$

**Step 5:** Calculate the standard deviation.

The standard deviation is calculated to measure the dispersion of each criterion in TSFM from reference point.

13 $$\begin{aligned} SD^{Pe}=\sqrt{\frac{1}{f-1} \sum \limits _{\mathfrak {b}=1}^{g} {(\tilde{l}_{\mathfrak {a}\mathfrak {b}}-M^{Pe})}^2}, \ \text {where} \ \mathfrak {a}=1,2,\ldots ,f, \ \mathfrak {b}=1,2,\ldots ,g. \end{aligned}$$

$$\begin{aligned} \ \ \ \ \ \ \ \ \ \ \ \mathfrak {C}_1 \ \mathfrak {C}_2 \ \cdots \ \mathfrak {C}_g \\ SD^{Pe}=[\tilde{s}_1 \ \tilde{s}_2 \ \cdots \ \tilde{s}_g] \end{aligned}$$

where $$\tilde{s}_b = \big ((x_{\mathfrak {b}}, y_{\mathfrak {b}}, z_{\mathfrak {b}}); \ell _{\mathfrak {b}}(h), \wp _{\mathfrak {b}}(h), \partial _{\mathfrak {b}}(h) \big ); \ \mathfrak {b}=1,2,\ldots ,g.$$

**Step 6:** Demonstrate the standard matrix $({\widetilde{SM}}^{Pe})$.

The standard score of each alternative is measured using Equation (14).

14 $$\begin{aligned} {\widetilde{SM}}^{Pe}=\frac{\tilde{l}_{\mathfrak {a}\mathfrak {b}}-M^{Pe}}{SD^{Pe}} \end{aligned}$$

where $${\widetilde{SM}}^{Pe}={[{\tilde{s}}^{Pe}_{\mathfrak {a}\mathfrak {b}}]}_{f \times g}; \ {\tilde{s}}^{Pe}_{\mathfrak {a}\mathfrak {b}}=\big ((x_{\mathfrak {a}\mathfrak {b}}, y_{\mathfrak {a}\mathfrak {b}}, z_{\mathfrak {a}\mathfrak {b}}); \ell _{\mathfrak {a}\mathfrak {b}}(s), \wp _{\mathfrak {a}\mathfrak {b}}(s), \partial _{\mathfrak {a}\mathfrak {b}}(s) \big ); \ \mathfrak{a}=1,2,\ldots ,f;\ \mathfrak{b}=1,2,\ldots ,g.$$.

**Step 7:** Determine the normalized matrix $({\widetilde{NM}}^{Pe})$.

The standard matrix ${\widetilde{SM}}^{Pe}$ is normalized using Equation (15).

15 $$\begin{aligned} {\widetilde{NM}}^{Pe}=\frac{{\tilde{s}}^{Pe}_{\mathfrak {a}\mathfrak {b}} - \min ({\tilde{s}}^{Pe}_{\mathfrak {a}\mathfrak {b}})}{{\max ({\tilde{s}}^{Pe}_{\mathfrak {a}\mathfrak {b}})} - \min ({\tilde{s}}^{Pe}_{\mathfrak {a}\mathfrak {b}})} \end{aligned}$$

where $${\widetilde{NM}}^{Pe}={[{\tilde{n}}^{Pe}_{\mathfrak {a}\mathfrak {b}}]}_{f \times g}; \ {\tilde{n}}^{Pe}_{\mathfrak {a}\mathfrak {b}}=\big ((x_{\mathfrak {a}\mathfrak {b}}, y_{\mathfrak {a}\mathfrak {b}}, z_{\mathfrak {a}\mathfrak {b}}); \ell _{\mathfrak {a}\mathfrak {b}}(n), \wp _{\mathfrak {a}\mathfrak {b}}(n), \partial _{\mathfrak {a}\mathfrak {b}}(n) \big );\ \mathfrak{a}=1,2,\ldots ,f;\ \mathfrak{b}=1,2,\ldots ,g$$; $${\widetilde{NM}}^{Pe} \in [0,1]$$. 

**Step 8:** Determine the defuzzified performance matrix $({O}^{Pe})$.

The ${\widetilde{NM}}^{Pe}$ is defuzzified to obtain the performance crisp score of the alternatives and criteria using Equation (6). The defuzzified matrix ${O^{Pe}}={[o_{\mathfrak {a}\mathfrak {b}}]}_{f \times g}; \ [o_{\mathfrak {a}\mathfrak {b}}]; \ \mathfrak {a}=1,2,\ldots ,f;\ \mathfrak {b}=1,2,\ldots ,g$.

When $'e'$ linguistic performance matrices (LPeMs) are designed, the aggregation matrix is obtained using Equation (16).

16 $$\begin{aligned} Q^{Pe}=\frac{\sum \limits _{c=1}^e {o}_{\mathfrak {a}\mathfrak {b}}^{c}}{e} \end{aligned}$$

where $${{Q}^{Pe}}={[{{q}}_{\mathfrak {a}\mathfrak {b}}]}_{f \times g}=[{q}_{\mathfrak {a}\mathfrak {b}}]$$; $$\mathfrak {a}=1,2,\ldots ,f; \ \mathfrak {b}=1,2,\ldots ,g$$.

**Step 9:** Determine the association between the performance and preference matrix.

**Sub-step 9(i)** Let us consider the preference vector as input vector. So, the input vector is $Y^{0}=[y_1^0 \ y_2^0 \ \cdots \ y_g^0]$. The input vector $Y^0$ is passed on the transposed defuzzified matrix ${(O^{Pe})^{T}}=[o_{\mathfrak {ba}}]$, represented in Equations (17) and update the input vector i.e., $Y^{\alpha }=[y_1^{\alpha } \ y_2^{\alpha } \ \cdots \ y_g^{\alpha }]$.

17 $$\begin{aligned} \widehat{X}^{\alpha }=[{\widehat{x}}_{\mathfrak {a}}^{\alpha }] = \sum \limits _{\mathfrak {b}=1}^{g} y^{\alpha }_{\mathfrak {b}} \times {o_{\mathfrak {b}\mathfrak {a}}}, \ \text {where} \ \alpha =1,2,\ldots ,n; \ n \ {\text {is the number of iterations}}. \end{aligned}$$

Apply the activation function using sigmoidal function on the resultant vector $\widehat{X}^{\alpha }$ as in Equation (18).

18 $$\begin{aligned} {X}^{\alpha }=[x_b^{\alpha }]= f({\widehat{x}}_{\mathfrak {a}}^{\alpha })=\frac{1}{1+e^{- \lambda \widehat{x}_{\mathfrak {a}}^{\alpha }}} \end{aligned}$$

where $$\mathfrak {a}=1,2,\ldots ,f$$ and $$\lambda>0$$.

**Sub-step 9(ii)** Now, pass the resultant vector *f* into ${O^{Pe}}=[o_{\mathfrak {ab}}]$ as in Equation (19).

19 $$\begin{aligned} \widehat{Y}^{\alpha }=[\widehat{y}_b^{\alpha }] = \sum \limits _{\mathfrak {a}=1}^{f} {x}_{\mathfrak {a}}^{\alpha } \times o_{\mathfrak {a}\mathfrak {b}}, \ \text {where} \ \mathfrak {b}=1,2,\ldots ,g; \ \alpha =1,2,\ldots ,n; \ n \ {\text {is number of iterations}}. \end{aligned}$$

Apply the activation function using the sigmoidal function on the resultant vector $\widehat{Y}^{\alpha }$ (Equation (20)).

20 $$\begin{aligned} Y^{\alpha }=[y_{\mathfrak {b}}^{\alpha }] = f(\widehat{y}_{\mathfrak {b}}^{\alpha })=\frac{1}{1+e^{- \lambda \widehat{y}_{\mathfrak {b}}^{\alpha }}} \end{aligned}$$

where $$\mathfrak {b}=1,2,\ldots ,g$$ and $$\lambda>0$$.

**Sub-step 9(iii)** Determine the converging point.

The iteration continues from sub-step 9(i) to sub-step 9(ii) untill the difference between the successive state vector reaches 0.001. i.e., $|Y^{\alpha + 1} - Y^{\alpha }| \le 0.001$ and $|X^{\alpha + 1} - X^{\alpha }| \le 0.001$.

**Step 10:** Determine the rank of alternatives.

Based on the converged $X^{\alpha }$ vector, the alternatives are graded $\big (R(B_{\mathfrak {a}})\big )$ in descending order.

**Segment III: Satisfactory measure**

This section evaluates the satisfaction level of the obtained ranks of each alternative.

**Step 1:** Construct the adjacency matrix.

The adjacency matrix ${P}={[{P}_{\mathfrak {a}\mathfrak {a}^{\prime }}]}_{f \times f}, \ \text {where} \ \mathfrak {a},\mathfrak {a}^{\prime }=1,2,\ldots ,f; \ \text {and} \ \mathfrak {a} \ne \mathfrak {a}^{\prime }$, is constructed based on the dominance score of alternatives.

**Step 2:** Determine the comparative function.

The difference of an each alternative is computed by comparing with its neighbourhood alternatives.

21 $$\begin{aligned} D(\mathfrak {d_a} - {\mathfrak {d_a}}^{\prime }) = \mathfrak {d_a} - {\mathfrak {d_a}}^{\prime }, \ \text {where} \ \mathfrak {a},\mathfrak {a}^{\prime }=1,2,\ldots ,f \text { and } \mathfrak {a} \ne \mathfrak {a}^{\prime } \end{aligned}$$

**Step 3:** Aggregate the comparative matrix.

Combine all the compared values of each alternatives.

22 $$\begin{aligned} Agg \big (D(\mathfrak {d_a} - {\mathfrak {d_a}}^{\prime })\big ) = {\sum \limits _{\begin{array}{c} {\mathfrak {a},\mathfrak {a}^{\prime }=1} \\ {\mathfrak {a} \ne \mathfrak {a}^{\prime }} \end{array}}^{f} \mathfrak {d_a} - {\mathfrak {d_a}}^{\prime }} \end{aligned}$$

**Step 4:** Calculate the positive and negative flows.

The $F^{+}$ and $F^{-}$ flows determines the positive and negative flows of the alternatives, respectively.

23 $$\begin{aligned} F^{+}_{\mathfrak {a}}= \frac{\sum \limits _{\mathfrak {a}=1}^{f} Agg \big (D(\mathfrak {d_a} - {\mathfrak {d_a}}^{\prime })\big )}{f-1} \end{aligned}$$

24 $$\begin{aligned} F^{-}_{\mathfrak {a}}=\frac{\sum \limits _{\mathfrak {a}=1}^{f} Agg \big (D(\mathfrak {d_a} - {\mathfrak {d_a}}^{\prime })\big )}{f-1} \end{aligned}$$

**Step 5:** Calculate the overall flow.

Using the positive and negative flows, the overall flow of each alternative is calculated.

25 $$\begin{aligned} F_{\mathfrak {a}}= F^{+}_{\mathfrak {a}} - F^{-}_{\mathfrak {a}}, \ \text {where} \ \mathfrak {a}=1,2,\ldots ,f. \end{aligned}$$

**Step 6:** Determine the rank of alternatives.

**Fig. 2:**
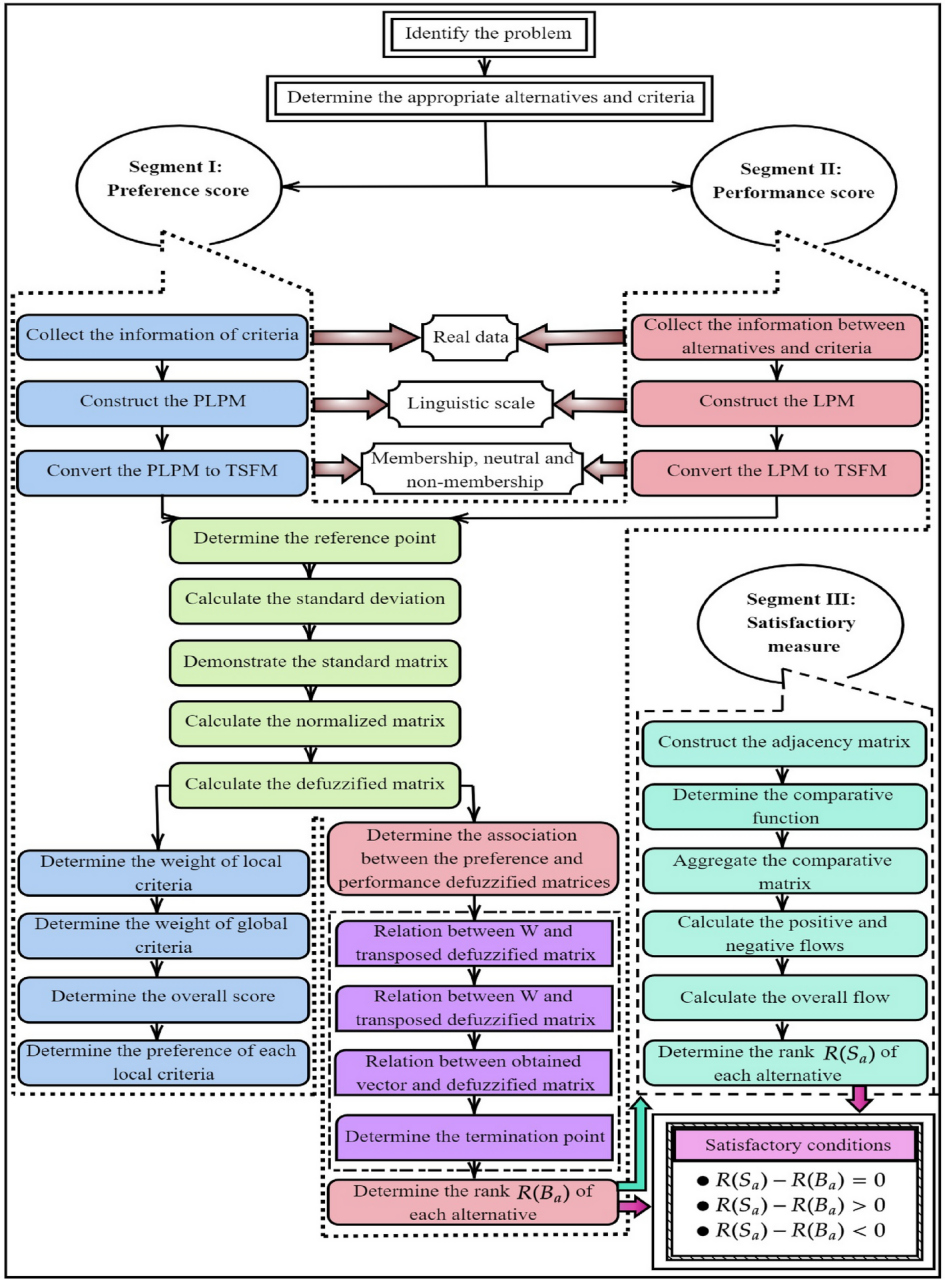
Graphical flow of APPSS method.

Through the values of overall flow, the alternatives are ranked $\big (R(S_{\mathfrak {a}})\big )$ in the descending order. The proposed method is graphically presented in Figure 2.

The satisfactory conditions are given below by comparing both the ranks of the alternative.

- If ${R(S_{\mathfrak {a}}) - R(B_{\mathfrak {a}})} = 0$, then the rank of alternatives in $R(B_{\mathfrak {a}})$ are the most optimal alternatives.
- If ${R(S_{\mathfrak {a}}) - R(B_{\mathfrak {a}})}> 0$, then the rank of alternatives in $R(B_{\mathfrak {a}})$ are the acceptable optimal alternatives.
- If ${R(S_{\mathfrak {a}}) - R(B_{\mathfrak {a}})} < 0$, then the rank of alternatives in $R(B_{\mathfrak {a}})$ are the less optimal alternatives.

## Illustration of the proposed method in finding the impact of COVID-19 in India

Since 2019, the global impact of the coronavirus has been significant, affecting a large number of people. It is significant to scrutinize the overall COVID-19 case count in heavily populated countries to mitigate the future complications. With India being the most populous country globally, this analysis focuses on the total number of COVID-19 cases affecting various age groups in India from 2020 to 2022.

**Fig. 3:**
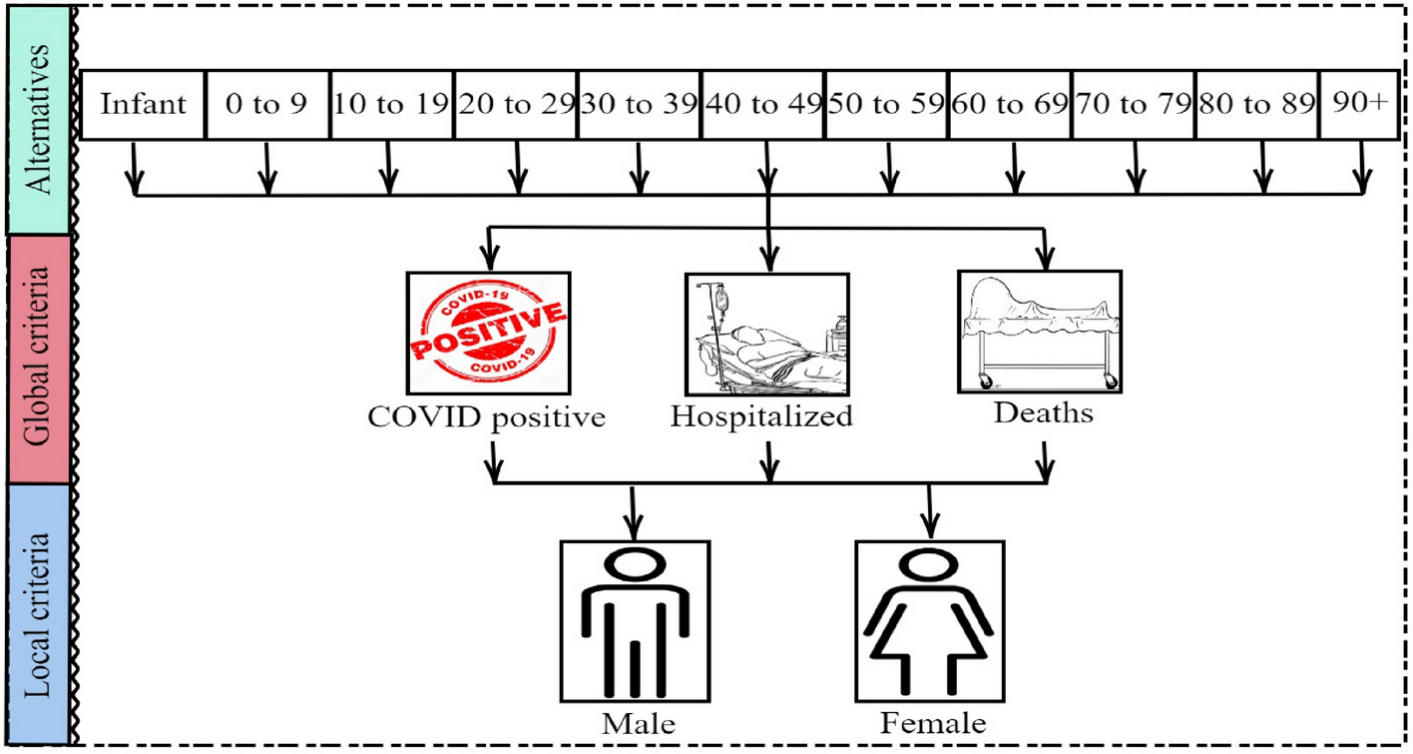
Alternatives and criteria.

The diverse age groups of the population are considered as alternatives, denoted as $\mathfrak {d_a}=\{\mathfrak {d}_1,\mathfrak {d}_2,\ldots ,\mathfrak {d}_{11}\}$, and these alternatives are evaluated based on three global criteria. Each global criterion comprises two local criteria, denoted as $\mathfrak {C_b}=\{\mathfrak {C}_1,\mathfrak {C}_2,\ldots ,\mathfrak {C}_6\}$. The considered alternatives and criteria are provided in Figure 3.

**Segment I:**

This segment focuses on assessing the significance of the criteria used in the analysis. It involves evaluating each criterion to determine its impact and contribution to the overall evaluation process. Additionally, this process ensures that the criteria are appropriately weighted, reflecting their respective importance in influencing the alternatives.

**Step 1:**The global and local criteria based on the problem and their interrelation between local criteria are collected from the article^55^.

**Step 2:** According to expert opinion, the PLPM matrix (Table 1) is formulated for the criteria based on the collected information.

**Table 1:** Pairwise linguistic preference matrix.

Criteria	COVID-19 positive		Hospitalized		Deaths	
Sub-criteria	Male ($\mathfrak {C_1}$)	Female ($\mathfrak {C_2}$)	Male ($\mathfrak {C_3}$)	Female ($\mathfrak {C_4}$)	Male ($\mathfrak {C_5}$)	Female ($\mathfrak {C_6}$)
$\mathfrak {C_1}$	-	$\mathcal {K}_4$	$\mathcal {K}_6$	$\mathcal {K}_4$	$\mathcal {K}_8$	$\mathcal {K}_4$
$\mathfrak {C_2}$	$\mathcal {K}_5$	-	$\mathcal {K}_5$	$\mathcal {K}_5$	$\mathcal {K}_6$	$\mathcal {K}_4$
$\mathfrak {C_3}$	$\mathcal {K}_6$	$\mathcal {K}_5$	-	$\mathcal {K}_7$	$\mathcal {K}_5$	$\mathcal {K}_5$
$\mathfrak {C_4}$	$\mathcal {K}_6$	$\mathcal {K}_4$	$\mathcal {K}_7$	-	$\mathcal {K}_6$	$\mathcal {K}_7$
$\mathfrak {C_5}$	$\mathcal {K}_7$	$\mathcal {K}_4$	$\mathcal {K}_6$	$\mathcal {K}_5$	-	$\mathcal {K}_4$
$\mathfrak {C_6}$	$\mathcal {K}_6$	$\mathcal {K}_4$	$\mathcal {K}_5$	$\mathcal {K}_4$	$\mathcal {K}_8$	-

**Step 3:** The PLPM is transformed into TSFM through Table 2.

**Table 2:** Linguistic scale.

Linguistic terms	Triangular spherical fuzzy number
Ultra low $(\mathcal {K}_0)$	$\big ((0.00,0.05,0.10);0.95,0.05,0.10\big )$
Supreme low $(\mathcal {K}_1)$	$\big ((0.07,0.14,0.21);0.05,0.10,0.20\big )$
Very low $(\mathcal {K}_2)$	$\big ((0.18,0.22,0.26);0.85,0.20,0.25\big )$
Slightly low $(\mathcal {K}_3)$	$\big ((0.25,0.28,0.30);0.80,0.30,0.30\big )$
Low $(\mathcal {K}_4)$	$\big ((0.29,0.36,0.43);0.70,0.35,0.40\big )$
Intermediate $(\mathcal {K}_5)$	$\big ((0.38,0.45,0.52);0.65,0.40,0.50\big )$
High $(\mathcal {K}_6)$	$\big ((0.50,0.56,0.62);0.55,0.50,0.60\big )$
Slightly high $(\mathcal {K}_7)$	$\big ((0.61,0.68,0.75);0.45,0.40,0.70\big )$
Very high $(\mathcal {K}_8)$	$\big ((0.73,0.83,0.88);0.25,0.20,0.75\big )$
Supreme high $(\mathcal {K}_9)$	$\big ((0.85,0.94,0.98);0.10,0.10,0.85\big )$
Ultra high $(\mathcal {K}_{10})$	$\big ((0.95,0.985,1.00);0.05,0.05,0.95\big )$

**Step 4:** The reference point (average) for TSFM is computed using Equation (2).

**Step 5:** The standard deviation for TSFM is determined using Equation (3).

**Step 6:** Utilizing the computed mean and standard deviation, the standard matrix is assessed by comparing each alternative with each criterion using Equation (4).

**Step 7:** The standard matrix undergoes normalization using Equation (5), which makes the performance criteria in the interval [0,1].

**Step 8:** The normalization matrix is defuzzified to yield the crisp score using Equation (6). The defuzzified values of criteria are presented in Table 3.

**Table 3:** Defuzzified matrix of criteria.

	$\mathfrak {C_1}$	$\mathfrak {C_2}$	$\mathfrak {C_3}$	$\mathfrak {C_4}$	$\mathfrak {C_5}$	$\mathfrak {C_6}$
$\mathfrak {C_1}$	0.000	0.712	0.742	0.430	0.595	0.430
$\mathfrak {C_2}$	0.554	0.000	0.554	0.582	0.601	0.430
$\mathfrak {C_3}$	0.742	0.976	0.000	0.881	0.449	0.582
$\mathfrak {C_4}$	0.742	0.712	0.831	0.000	0.601	0.881
$\mathfrak {C_5}$	0.831	0.712	0.742	0.582	0.000	0.430
$\mathfrak {C_6}$	0.742	0.712	0.554	0.430	0.595	0.000

**Step 9:** The weight of each local criteria is calculated using Equation (8).

**Step 10:** Using the local criteria weight, the global criteria weight is measured using Equation (9).

**Step 11:** The overall weight is computed by assigning equal importance to both local and global criteria in Equation (10). Therefore, the parameter $\alpha$ is set to 0.5.

**Step 12:** The preference vector is measured using Equation (11). The resulting preference vector is:

$$\begin{aligned} \ \ \ \ \ \ \ \mathfrak {C_1} \ \ \ \ \ \ \mathfrak {C_2} \ \ \ \ \ \ \mathfrak {C_3} \ \ \ \ \ \mathfrak {C_4} \ \ \ \ \ \mathfrak {C_5} \ \ \ \ \ \mathfrak {C_6} \\ W=[0.149 \ 0.142 \ 0.189 \ 0.193 \ 0.168 \ 0.159] \end{aligned}$$

**Segment II:**

**Step 1:** The information regarding the alternatives and criteria presented in Table 4 is sourced from the article by Singh et al.^55^.

**Table 4:** Data of the alternatives and criteria.

	$\mathfrak {C_1}$	$\mathfrak {C_2}$	$\mathfrak {C_3}$	$\mathfrak {C_4}$	$\mathfrak {C_5}$	$\mathfrak {C_6}$
$\mathfrak {d_1}$	32433	25500	2795	2062	97	77
$\mathfrak {d_2}$	663464	566640	53118	44665	272	153
$\mathfrak {d_3}$	1859655	1493623	141900	106762	595	505
$\mathfrak {d_4}$	4863748	3424344	381877	247425	3562	2189
$\mathfrak {d_5}$	5574392	3392564	484538	267434	12324	4833
$\mathfrak {d_6}$	4220769	2750252	387151	226250	24083	11251
$\mathfrak {d_7}$	3323505	2327632	311917	197033	39339	20921
$\mathfrak {d_8}$	2191686	1607215	211645	150805	50661	28396
$\mathfrak {d_9}$	1049700	714205	103857	67464	39533	19875
$\mathfrak {d_{10}}$	302475	218457	27561	18059	16409	7792
$\mathfrak {d_{11}}$	41778	39051	3638	3405	2509	1533

**Step 2:** The collected data displays diverse ranges, requiring for fuzzification. Here, the number of cases “$\le$1000%” is denoted as $(\mathcal {K}_0)$, “1000 - 25000” as $(\mathcal {K}_1)$, “25001 - 100000” as $(\mathcal {K}_2)$, “100001 - 250000” as $(\mathcal {K}_3)$, “250001 - 500000” as $(\mathcal {K}_4)$, “500001 - 1000000” as $(\mathcal {K}_5)$, “1000001 - 2000000” as $(\mathcal {K}_6)$, “2000001 - 3000000” as $(\mathcal {K}_7)$, “3000001 - 4000000” as $(\mathcal {K}_8)$, “4000001 - 5000000” as $(\mathcal {K}_9)$, and “$\ge$5000000%” as $(\mathcal {K}_{10})$. Through this process, the obtained data is transformed into the linguistic performance matrix (LPeM).

**Step 3:** The LPeM is transmuted into TSFM using Table 2.

**Step 4:** The reference point for each criteria is calculated using Equation (12).

**Step 5:** The standard deviation is determined to assess the dispersion of the collected data from the mean, as per Equation (13).

**Step 6:** The standard matrix is evaluated through the attained mean and standard deviation through Equation (14).

**Step 7:** The standard matrix is normalized using Equation (15).

**Step 8:** The normalized matrix is defuzzified using Equation (16).

**Step 9:** The relationship between the performance and preference matrices is measured.

**Sub-step 9(i):** The input vector (preference vector) and transposed defuzzified matrix are multiplied, resulting in the $\widehat{x}_{\mathfrak {a}}$ values. The $\widehat{x}_{\mathfrak {a}}$ values is updated through the sigmoidal function to get $x_{\mathfrak {a}}^{\alpha }$ using Equation (19).

**Sub-step 9(ii):** Then, the updated $x_{\mathfrak {a}}^{\alpha }$ vector is multiplied with defuzzified matrix to evaluate $\widehat{y}_{\mathfrak {b}}$ values. Now, the values of $\widehat{y}_{\mathfrak {b}}$ is updated using the sigmoidal function to get $Y_{\mathfrak {b}}^{\alpha }$ as described in Equation (20). The updated $Y_{\mathfrak {b}}$ vector is then normalized to achieve convergence with minimal iterations.

**Sub-step 9(iii):** The convergence are identified for the vectors ${X}^{\alpha }$ at their respective equilibrium points. Specifically, ${X}^{\alpha }$ converges to ${X}^{\alpha +1}$ in the $3^{rd}$ and $4^{th}$ iterations.

**Fig. 4:**
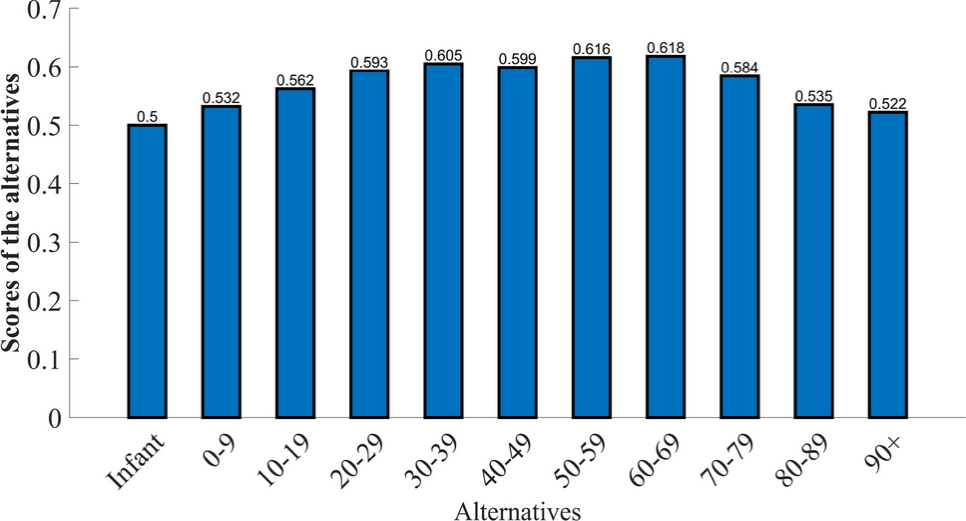
Scores of the alternatives.

**Step 10:** Then, the converged points of *X* vector (alternatives) provided in Table 5 are ranked $(R_{B})$ in descending order. Figure 4 represents the scores of the alternatives.

**Table 5:** Rank $(R_{B})$ of the alternatives.

Alternatives	$\mathfrak {d_1}$	$\mathfrak {d_2}$	$\mathfrak {d_3}$	$\mathfrak {d_4}$	$\mathfrak {d_5}$	$\mathfrak {d_6}$	$\mathfrak {d_7}$	$\mathfrak {d_8}$	$\mathfrak {d_9}$	$\mathfrak {d_{10}}$	$\mathfrak {d_{11}}$
*X* converging point	0.500	0.532	0.562	0.593	0.605	0.599	0.616	0.618	0.584	0.535	0.522
Rank $(R_{B})$	11	9	7	5	3	4	2	1	6	8	10

**Segment III:**

**Step 1:** To assess the satisfactory measure of outcomes, the adjacency matrix *P* is formed based on the scores of the alternatives. The relationships between alternatives in matrix *P* are represented in binary, where a value 1 indicates dominance when one alternative’s score surpasses the other, and a value 0 indicates no dominance.

**Step 2:** The disparity between each alternative is calculated to identify the preferred alternative, as indicated in Equation (21).

**Step 3:** The cumulative impact of each alternative, whether preferred or not, is assessed by measuring the two distinct flows: one leaving (positive) and the other entering (negative).

**Step 4:** The overall flow for each alternative is calculated by subtracting the positive and negative flows of each alternative. Subsequently, the overall flow score $(R_{S})$ is ranked based on the descending order. The rank $(R_{S})$ of the alternatives are presented in Table 6.

**Table 6:** Rank $(R_{S})$ of the alternatives.

Alternatives	$\mathfrak {d_1}$	$\mathfrak {d_2}$	$\mathfrak {d_3}$	$\mathfrak {d_4}$	$\mathfrak {d_5}$	$\mathfrak {d_6}$	$\mathfrak {d_7}$	$\mathfrak {d_8}$	$\mathfrak {d_9}$	$\mathfrak {d_{10}}$	$\mathfrak {d_{11}}$
Overall flow (*F*)	0.2	−7	−3	1	5	3	4.8	9	−1	−5	−7
Rank $(R_{S})$	6	10	8	5	2	4	3	1	7	9	10

Now, the satisfaction level is assessed for all alternatives by subtracting $(R_{B})$ from $(R_{S})$. The satisfactory value of each alternative are presented in Table 7. The alternatives achieving a value of 0 are considered as age groups highly or lowly impacted by COVID.

**Table 7:** Satisfactory value.

Alternatives	$\mathfrak {d_1}$	$\mathfrak {d_2}$	$\mathfrak {d_3}$	$\mathfrak {d_4}$	$\mathfrak {d_5}$	$\mathfrak {d_6}$	$\mathfrak {d_7}$	$\mathfrak {d_8}$	$\mathfrak {d_9}$	$\mathfrak {d_{10}}$	$\mathfrak {d_{11}}$
Rank $(R_{B})$	11	9	7	5	3	4	2	1	6	8	10
Rank $(R_{S})$	6	10	8	5	2	4	3	1	7	9	10
$R_{S}-R_{B}$	−5	1	1	0	−1	0	1	0	1	1	0

## Results and discussion

By scrutinizing various fuzzy MCDM methods, it became evident that the existing MCDM approach lacks a comprehensive examination of the association between preference and performance. To address this gap, this study introduced the fuzzy APPSS method by utilizing the BAM concept to assess preference and performance along with their respective satisfactory score. The existing methods derive maximum values through utility degree, whereas the proposed APPSS method calculated the maximum value for each alternative by identifying the converging point of each alternative. These converging points were ranked, and the level of satisfaction was determined by constructing an adjacency matrix based on the converged score using the dominance concept. If the score of alternative $(A_i)$ dominated the score of alternative $(A_j)$ where $i \ne j$, the corresponding entry in the adjacency matrix was denoted as 1; otherwise, it was denoted as 0. The overall scores of alternatives were measured through the positive and negative flows within the adjacency matrix and subsequently, they were ranked. The final ranking of alternatives was determined through $R_{B}$. This conclusion was analyzed using a satisfactory measure, and the alternatives were again ranked using $R_{S}$. The obtained ranks of alternatives were verified using the provided three satisfactory conditions by subtracting $R_{S}$ and $R_{B}$. For instance, alternative $\mathfrak {d_8}$ (60–69) got the same rank in both $R_{B}$ and $R_{S}$ where its satisfactory value is 0, and the age group is higher vulnerability of COVID than other age groups. Conversely, alternative $\mathfrak {d_1}$ (Infant) exhibits a disparity in ranking within both $R_{B}$ and $R_{S}$, yielding a satisfactory value of −5, suggesting lower susceptibility to COVID compared to other age groups.

Based on these findings, the most impacted age groups within the specified COVID category were $\mathfrak {d_8}(60-69)>\mathfrak {d_7}(50-59)>\mathfrak {d_5}(30-39)$. These findings are corroborated by the study conducted in article^56^, where it was observed that the mortality rate among individuals aged 60–69 were increased during the initial lockdown and also in the first and second waves of the COVID-19 pandemic compared to other age groups. Furthermore, a report published by The Indian Express on March 16, 2024, (https://indianexpress.com/article/india/more-than-half-of-covid-19-deaths-in-age-group-of-50-to-69-years-maharashtra-govt-analysis-6577946/) revealed that the age group most susceptible to severe COVID-19 are between 50 to 69 years. Therefore, the comparison of the obtained result with the real data confirms the efficacy of the proposed method.

### Sensitivity analysis

**Fig. 5:**
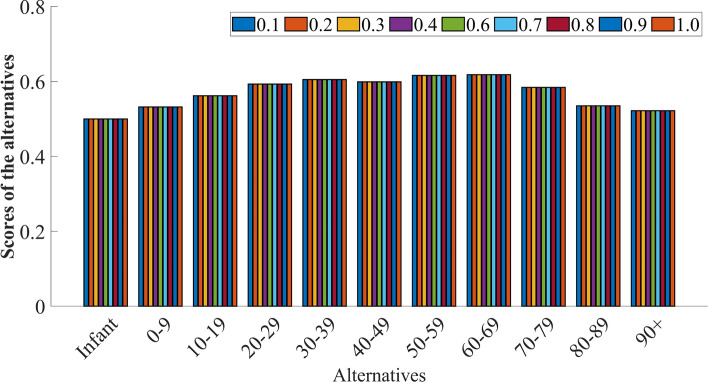
Sensitivity analysis.

Sensitivity analysis aids in evaluating the robustness of the decision-making process by tuning the input parameters in the model. So, it helps to identify which inputs have the most significant impact on the final decision. Here, the impact of different $\alpha$ values on both local and global criteria is scrutinized to observe their effect on the outcomes of the alternatives. In the proposed approach, $\alpha$ is fixed at 0.5 to ensure equal weighting of local and global criteria. During the sensitivity analysis, $\alpha$ is varied from 0.0 to 1.0. This range allows us to assess how shifting the emphasis between local and global criteria influences the overall rankings and performance of the alternatives. Specifically, when $\alpha$ is 0.0, the weighting process considers only the global criteria, and when $\alpha$ is 1.0, the weighting process considers only the local criteria. However, the different $\alpha$ values do not affect the ranking of the alternatives are depicted in Table 8 and Figure 5.

**Table 8:** Sensitivity analysis.

$\alpha$ values	Ranking order
0.0	$\mathfrak {d_8}>\mathfrak {d_7}>\mathfrak {d_5}>\mathfrak {d_6}>\mathfrak {d_4}>\mathfrak {d_9}>\mathfrak {d_3}>\mathfrak {d_{10}}>\mathfrak {d_2}>\mathfrak {d_{11}}>\mathfrak {d_1}$
0.1	$\mathfrak {d_8}>\mathfrak {d_7}>\mathfrak {d_5}>\mathfrak {d_6}>\mathfrak {d_4}>\mathfrak {d_9}>\mathfrak {d_3}>\mathfrak {d_{10}}>\mathfrak {d_2}>\mathfrak {d_{11}}>\mathfrak {d_1}$
0.2	$\mathfrak {d_8}>\mathfrak {d_7}>\mathfrak {d_5}>\mathfrak {d_6}>\mathfrak {d_4}>\mathfrak {d_9}>\mathfrak {d_3}>\mathfrak {d_{10}}>\mathfrak {d_2}>\mathfrak {d_{11}}>\mathfrak {d_1}$
0.3	$\mathfrak {d_8}>\mathfrak {d_7}>\mathfrak {d_5}>\mathfrak {d_6}>\mathfrak {d_4}>\mathfrak {d_9}>\mathfrak {d_3}>\mathfrak {d_{10}}>\mathfrak {d_2}>\mathfrak {d_{11}}>\mathfrak {d_1}$
0.4	$\mathfrak {d_8}>\mathfrak {d_7}>\mathfrak {d_5}>\mathfrak {d_6}>\mathfrak {d_4}>\mathfrak {d_9}>\mathfrak {d_3}>\mathfrak {d_{10}}>\mathfrak {d_2}>\mathfrak {d_{11}}>\mathfrak {d_1}$
0.6	$\mathfrak {d_8}>\mathfrak {d_7}>\mathfrak {d_5}>\mathfrak {d_6}>\mathfrak {d_4}>\mathfrak {d_9}>\mathfrak {d_3}>\mathfrak {d_{10}}>\mathfrak {d_2}>\mathfrak {d_{11}}>\mathfrak {d_1}$
0.7	$\mathfrak {d_8}>\mathfrak {d_7}>\mathfrak {d_5}>\mathfrak {d_6}>\mathfrak {d_4}>\mathfrak {d_9}>\mathfrak {d_3}>\mathfrak {d_{10}}>\mathfrak {d_2}>\mathfrak {d_{11}}>\mathfrak {d_1}$
0.8	$\mathfrak {d_8}>\mathfrak {d_7}>\mathfrak {d_5}>\mathfrak {d_6}>\mathfrak {d_4}>\mathfrak {d_9}>\mathfrak {d_3}>\mathfrak {d_{10}}>\mathfrak {d_2}>\mathfrak {d_{11}}>\mathfrak {d_1}$
0.9	$\mathfrak {d_8}>\mathfrak {d_7}>\mathfrak {d_5}>\mathfrak {d_6}>\mathfrak {d_4}>\mathfrak {d_9}>\mathfrak {d_3}>\mathfrak {d_{10}}>\mathfrak {d_2}>\mathfrak {d_{11}}>\mathfrak {d_1}$
1.0	$\mathfrak {d_8}>\mathfrak {d_7}>\mathfrak {d_5}>\mathfrak {d_6}>\mathfrak {d_4}>\mathfrak {d_9}>\mathfrak {d_3}>\mathfrak {d_{10}}>\mathfrak {d_2}>\mathfrak {d_{11}}>\mathfrak {d_1}$

### Comparative analysis

**Fig. 6:**
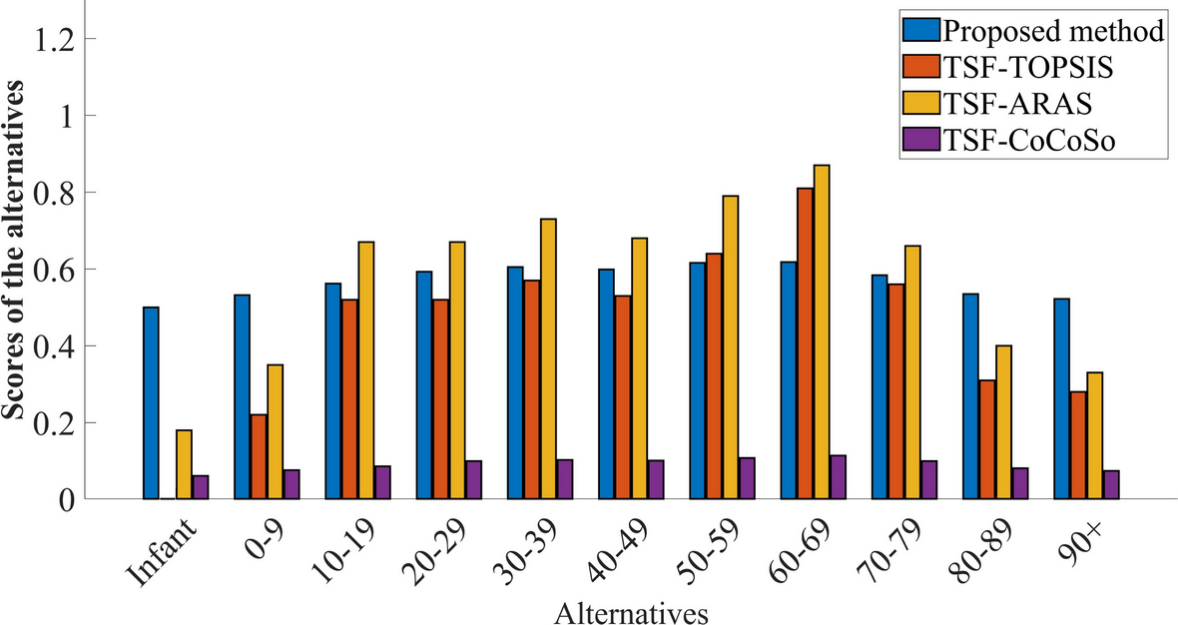
Comparative analysis.

The proposed method is validated by comparative analysis through three distinct MCDM techniques: TSF-TOPSIS, TSF-ARAS, and TSF-CoCoSo. In these methods, the criteria weights are determined using the entropy technique. The results (Table 9) demonstrate that the optimal alternatives identified by the proposed method are consistent with those identified by existing approaches and represented in Figure 6. Despite achieving similar results, the proposed method employs satisfactory scores to indicate the level of satisfaction with each alternative’s performance on criteria. Such a nuanced evaluation provides a more realistic assessment of alternative performances compared to conventional approaches. This congruence demonstrates the robustness and effectiveness of the proposed method, confirming its validity and reliability in producing consistent and accurate outcomes.

**Table 9:** Comparative analysis.

Fuzzy MCDM models	$\mathfrak {d_1}$	$\mathfrak {d_2}$	$\mathfrak {d_3}$	$\mathfrak {d_4}$	$\mathfrak {d_5}$	$\mathfrak {d_6}$	$\mathfrak {d_7}$	$\mathfrak {d_8}$	$\mathfrak {d_9}$	$\mathfrak {d_{10}}$	$\mathfrak {d_{11}}$	Ranking order
Proposed method	0.500	0.532	0.562	0.593	0.605	0.599	0.616	0.618	0.584	0.535	0.522	$\mathfrak {d_8}>\mathfrak {d_7}>\mathfrak {d_5}>\mathfrak {d_6}>\mathfrak {d_4}>\mathfrak {d_9}>\mathfrak {d_3}>\mathfrak {d_{10}}>\mathfrak {d_2}>\mathfrak {d_{11}}>\mathfrak {d_1}$
TSF-TOPSIS	0.00	0.22	0.34	0.52	0.57	0.53	0.64	0.81	0.56	0.31	0.28	$\mathfrak {d_8}>\mathfrak {d_7}>\mathfrak {d_5}>\mathfrak {d_6}>\mathfrak {d_4}>\mathfrak {d_9}>\mathfrak {d_3}>\mathfrak {d_{10}}>\mathfrak {d_{11}}>\mathfrak {d_2}>\mathfrak {d_1}$
TSF-ARAS	0.18	0.35	0.48	0.67	0.73	0.68	0.79	0.87	0.66	0.40	0.33	$\mathfrak {d_8}>\mathfrak {d_7}>\mathfrak {d_5}>\mathfrak {d_6}>\mathfrak {d_4}>\mathfrak {d_9}>\mathfrak {d_3}>\mathfrak {d_{10}}>\mathfrak {d_2}>\mathfrak {d_{11}}>\mathfrak {d_1}$
TSF-CoCoSo	0.060	0.075	0.085	0.100	0.103	0.101	0.108	0.114	0.100	0.080	0.073	$\mathfrak {d_8}>\mathfrak {d_7}>\mathfrak {d_5}>\mathfrak {d_6}>\mathfrak {d_9}>\mathfrak {d_4}>\mathfrak {d_3}>\mathfrak {d_{10}}>\mathfrak {d_2}>\mathfrak {d_{11}}>\mathfrak {d_1}$

Several advantages of the proposed method include its flexibility in handling both qualitative and quantitative data. It can also determine the significance of both global and local criteria. By utilizing BAM, the method effectively captures and evaluates the complex interdependencies among alternatives and criteria. Table 10 contains the components of fuzzy MCDM methods that utilized to solve the decision-making problem, which is clear that the proposed method differs and demonstrates its advancement from the existing approaches. This study has certain limitations as follows:

- The results are based on data collected from articles^55^ analyzing the impact of COVID-19 in India. These results could differ if data were sourced from different articles, portals or diverse field experts.
- Additionally, outcomes may vary across countries due to differing COVID-19 rates, which can influence the relationship between alternatives and criteria.

**Table 10:** Advantages of APPSS method.

Fuzzy MCDM models	Association between the alternatives and criteria	Pairwise comparison	Rank through converging point	Quantitative analysis	Qualitative analysis	Satisfactory level of each alternative
AHP	✗	✓	✗	$\LEFTcircle$	✓	✗
BWM	✗	✓	✗	✓	✗	✗
DEMATEL	✗	✓	✗	✓	✓	✗
TOPSIS	✓	✗	✗	✓	✓	✗
VIKOR	✓	✗	✗	✓	✓	✗
MABAC	✓	✗	✗	✓	✓	✗
APPSS	✓	✓	✓	✓	✓	✓

### Managerial implication

This study provides significant insights for advancing healthcare management and policy-making by addressing critical challenges posed by the COVID pandemic. By highlighting disparities in infection rates and identifying the most vulnerable age groups, it enables healthcare managers to prioritize resource allocation and address immediate needs, such as vaccination, nutritional support and mental health programs targeted to specific demographics. Additionally, the study emphasizes the importance of preparing for long-term health impacts, including post-viral syndromes, mental health challenges and the resurgence of diseases exacerbated by the pandemic. These insights empower healthcare systems to develop and implement preventive measures to reduce future healthcare challenges. The findings also provide robust evidence to public health policies for the development of strategies to mitigate similar crises in the future. Moreover, the proposed approach aids healthcare professionals to manage qualitative data and capable of addressing a wide range of real-world challenges by quantifying the level of satisfaction in the outcomes.

## Conclusion

The existing fuzzy models have been primarily emphasized on pairwise relationships and interactions among alternatives and criteria but lacked a concurrent comprehensive analysis of both preference and performance. To address this gap, this study was introduced a novel fuzzy APPSS method using BAM with satisfaction measures. The APPSS model extends its analytical scope by evaluating criteria preferences and the performance of both alternatives and criteria. It also balances the importance of global and local criteria when assessing weightage and used these weightage values to evaluate the performance of alternatives for each criterion through BAM. The BAM model identifies stronger and weaker associations between alternatives and criteria, where better-performing alternatives showed higher associations with beneficial criteria and lower associations with non-beneficial criteria. The reliability of outcomes was verified through a satisfactory score using an alternative adjacency matrix, where dominance relations were indicated by 1 and non-dominance by 0. The proposed model’s effectiveness was demonstrated by analyzing the impact of COVID-19 in India among various age groups from 2020 to 2022 within the TSFN framework. The scoring function aids in calculating the score of each fuzzy set. To measure the score of each TSFN, the scoring function was introduced using the graded mean integration method, utilized as a defuzzification technique. As the result, COVID-19 impact more on 60–69age group compared to others. The obtained results prove the proposed method’s efficiency and reliability. Furthermore, this approach can be extended by integrating fuzzy models with neural network concepts, focusing on various scoring functions, entropy, similarity measures and aggregation operators for higher order SFS^57^, bipolar fuzzy set, bipolar complex fuzzy set (BCFS)^58^, interval BCFS^59^, various operation on BCFS^60^. The BFS will extend the proposed method in two dimension (positive and negative). This integration aims to provide a more comprehensive analysis, enhancing the model’s versatility and applicability across different domains and selection decision-making scenarios.

## Data Availability

All data generated or analysed during this study are included in this published article and can be found in online at https://doi.org/10.3389/fpubh.2022.1027312.
